# Mapping philanthropic support of science

**DOI:** 10.1038/s41598-024-58367-2

**Published:** 2024-04-24

**Authors:** Louis M. Shekhtman, Alexander J. Gates, Albert-László Barabási

**Affiliations:** 1 Network Science Institute, Northeastern University, Boston, MA 02115 USA; 2 School of Data Science, University of Virginia, Charlottesville, VA 22904 USA; 3 Center for Cancer Systems Biology, Dana-Farber Cancer Institute, Boston, MA 02115 USA; 4 Department of Medicine, Brigham and Women’s Hospital, Harvard Medical School, Boston, MA 02115 USA; 5 Department of Network and Data Science, Central European University, Budapest, 1051 Hungary

**Keywords:** Complex networks, Scientific data

## Abstract

While philanthropic support for science has increased in the past decade, there is limited quantitative knowledge about the patterns that characterize it and the mechanisms that drive its distribution. Here, we map philanthropic funding to universities and research institutions based on IRS tax forms from 685,397 non-profit organizations. We identify nearly one million grants supporting institutions involved in science and higher education, finding that in volume and scope, philanthropy is a significant source of funds, reaching an amount that rivals some of the key federal agencies like the NSF and NIH. Our analysis also reveals that philanthropic funders tend to focus locally, indicating that criteria beyond research excellence play an important role in funding decisions, and that funding relationships are stable, i.e. once a grant-giving relationship begins, it tends to continue in time. Finally, we show that the bipartite funder-recipient network displays a highly overrepresented motif indicating that funders who share one recipient also share other recipients and we show that this motif contains predictive power for future funding relationships. We discuss the policy implications of our findings on inequality in science, scientific progress, and the role of quantitative approaches to philanthropy.

## Introduction

Since the emergence of a US federal funding system for research following World War II^1^, public sources of funding have failed to keep up with the growing demands of fundamental and applied research^2^. This contrasts with some other countries, such as Finland and Norway where government funding has continued to rise into the twenty-first century^3^. Despite this lack of increased government support, the US is fairly unique compared to other countries in that US Science has increasingly benefited from the support of private philanthropy, contributing up to 44% of basic research funding at US universities in 2016^4,5^. Philanthropy has also been credited for high-impact outcomes such as supporting the work of Chemistry Nobel Prize recipients Frances Arnold and Jennifer Doudna^6^. While the patterns characterizing government funding are closely monitored both in the US^7–11^ and around the world^12,13^, and are the subject of spirited policy debates, our understanding of the philanthropic ecosystem is often limited to summary statistics, case studies, or the largest gifts, and even these are not collected systematically, so studies must rely on media mentions or public gift announcements^4,14–19^. This narrow focus prohibits a quantitative understanding of the complete spectrum of philanthropic support for scientific institutions and is thus unable to identify systematic patterns that arise. Philanthropists and the community have become increasingly aware of these obstacles and have begun calling for increased research, both into science philanthropy and into philanthropic funding more generally^20–22^. The obstacles towards a quantitative understanding of philanthropy have primarily been rooted in data availability: while all details pertaining to federal funding are public and accessible for research purposes, we lack a similar transparency when it comes to philanthropic grant-giving.

Data access has fortunately improved recently thanks to changes by the Internal Revenue Service (IRS), who has made machine-readable Form 990 tax data available to the public^23^. This tax form is filed by all US non-profits and foundations (except churches), and contains information on the organization’s revenue, expenditures, executive leadership^24^, mission statement^25,26^, and more. This data resource is a windfall for researchers in philanthropic studies, a growing field that explores how nonprofit organizations operate and provides guidance to practitioners in the field. We used this resource to analyze over 3.6 million tax forms from 685,397 non-profit organizations in the United States between 2010 and 2019, extracting information about over 10 million grants (Fig. S1). The data allowed us to identify 69,675 nonprofit organizations involved in funding or performing scientific research, who together gave and received 926,124 grants totaling $208 billion. We find that funding offered by philanthropy to research institutions has reached $30B per year in recent years, rivaling the level of funding offered by the NIH (Fig. 2a).

Our analysis reveals important organizing principles for philanthropic funding. We find that while the government relies on a few large organizations to fund scientific research, the philanthropic ecosystem is extremely heterogenous and distributed, where a few large foundations coexist with many small funders. At the same time, the total funding that a recipient scientific organization receives from philanthropy is highly correlated to the amount they receive in NSF funding, suggesting philanthropy likely perpetuates previously observed scientific inequalities^27,28^. Indeed, the philanthropic ecosystem exhibits the similar levels of inequality throughout the past decade with the Gini Coefficient remaining above 0.8 (Fig. S7). Next, in contrast to centralized NSF funding with a mandate to distribute nationally, philanthropic funders have a strong preference for funding local organizations. We also show that the patterns of philanthropic funding are highly stable in time, with funders supporting the same recipients year after year. Finally, we find that philanthropic funders tend to share multiple recipients with one another suggesting that funders with similar aims tend to identify the same groups of recipients and that this network pattern contains predictive power for future grants.

### Philanthropic funding of science

**Figure 1:**
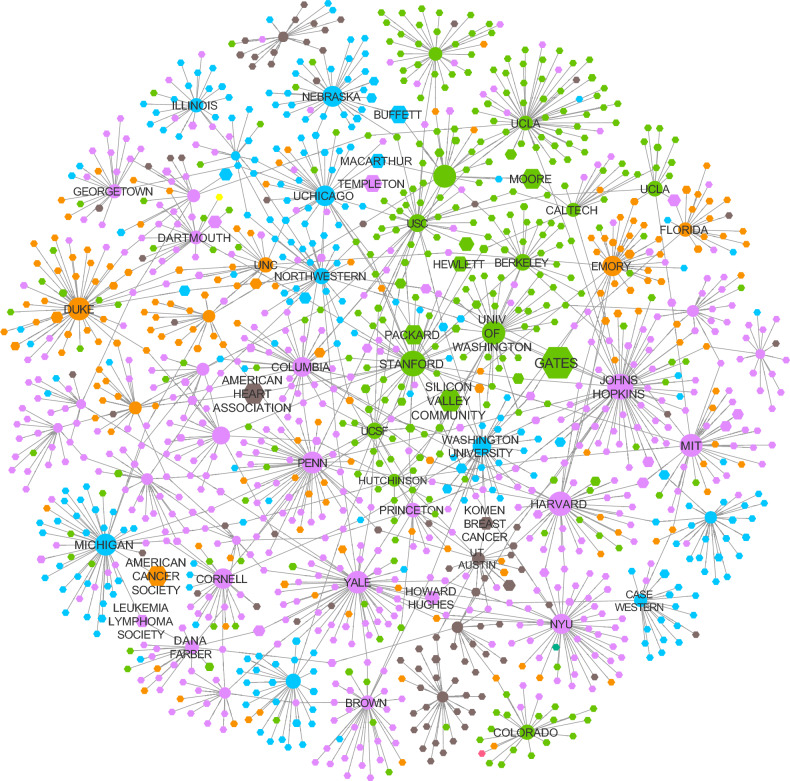
The philanthropic ecosystem of science. The network of funders and their top recipients. For each funder we maintained their top two recipients and then filtered to only include relationships worth over $1M over the period examined. We also removed donor advised funds and single-support foundations. The resulting network shows 55 recipients (circles) and 1254 donors (octagons) with 1422 grant relationships between them. Nodes are colored by region with purple being Northeast, blue being Midwest, green being west, brown being southwest, and orange being the south. We see that most donors have their top recipient/s in the same region, though those with multiple $1M + recipients at times have a top recipient in a different region. Figure generated with Cytoscape 3.10.

US-based nonprofit organizations are required to file Form 990, detailing their executive leadership, assets, cash flow, and layers of financial information, collected and publicly shared by the IRS. Nonprofit organizations self-report their area of activity under 26 categories, four of which have direct relevance to science: Social Science Research Institutes; Science and Technology Research Institutes; Medical Research; and Higher Education Institutions, together representing 30,351 organizations. We added to this list 3738 public universities and nonprofit organizations that receive grants on their behalf (see SI Section I). After identifying all grants received by these organizations, we arrive to 69,675 non-profit organizations who have donated or received funds that contribute to science. In Fig. 1, we show the network of 1254 funders who gave at least $1 M to one of 55 recipients. The nodes are colored by US region, unveiling the strong preference of funders for recipients in their same region.

We find a clear distinction between funders and recipients: 81% of the identified organizations only gave grants, 16% only received grants, and 3%, mainly universities, were involved in both giving and receiving. The fact that the number of funders significantly exceeds the number of recipients is somewhat unexpected, given the difficulty many organizations report in attracting funding^29^. However, it is worth noting that each university and many research institutions contain multiple departments and research groups that independently seek funding. Thus, rather than evidence of a plethora of funding opportunities available for scientists, the imbalance between donor and recipient organizations reflects the fact that most research is carried out within a few large institutions, mainly universities, that provide an appropriate institutional framework for research and fundraising.

We find that most funders who contribute to science and research also contribute to other philanthropic causes including art, human services, other education (aside from higher education), and religion. To capture the diversity of focus across funding organizations, we identified 7,124,144 grants throughout all areas of philanthropy by funders who gave at least one grant to research institutions (science donors). We then identified for each funding organization the area to which it donates the largest amount of funds. Only 16% of science donors had an exclusive science focus and an additional 28% of science donors gave more to science than to any other area (Fig. S5b). Funders with a primary focus on science together account for 93% of all scientific philanthropy, suggesting that the bulk of science funding comes from organizations who have chosen it as their primary area of philanthropy. At the same time, we find several prominent funders who give to science, yet their primary focus area is elsewhere (Fig. S5). For example, the Annenberg Foundation primarily funds art organizations, and the Sherwood Foundation predominately funds primary and secondary education organizations, but both foundations also support scientific research related to art and primary education, respectively.

We also connected philanthropic funding to scientific papers by analyzing acknowledgements as they appear in the paper. To do so, we relied on the dimensions.ai database, allowing us to identify 42 funders classified as nonprofits. Notably this is far fewer than the thousands of funders that contribute to science and were identified in our study, highlighting that most philanthropic funders are never directly cited in scientific papers. The 42 funders that do appear are cited in 21,472 papers with a primary focus on Medicine, Health and Biology (Fig. S22).

**Figure 2:**
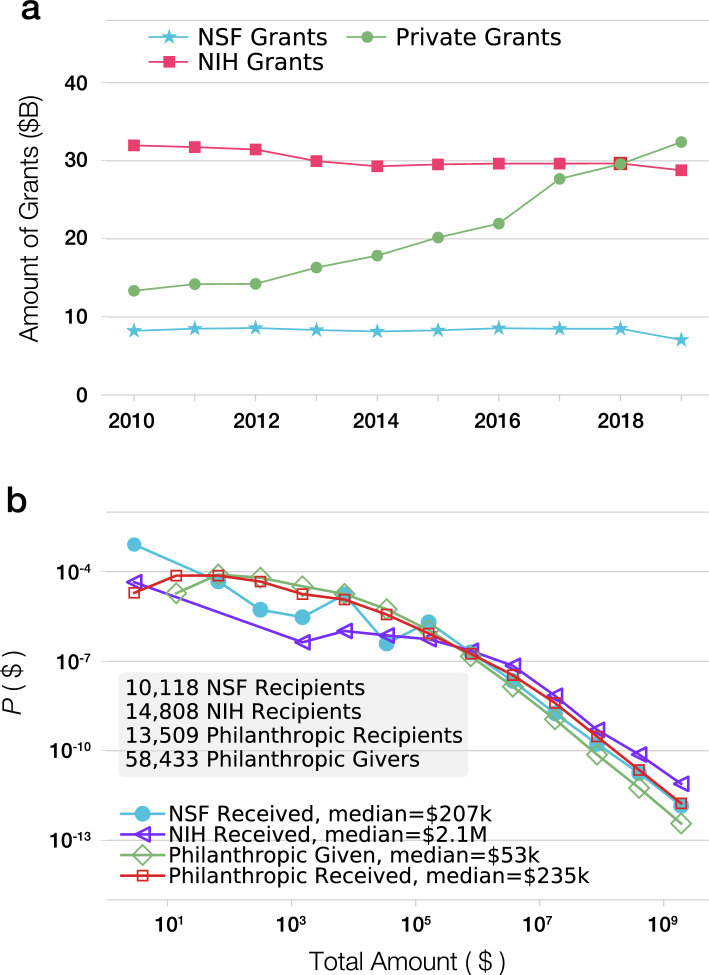
Philanthropic vs. federal support. (**a**) The amount of grants provided to institutions performing research by private nonprofit organizations has grown considerably over the past decade, surpassing the amount of grants given by the NSF and NIH^14^. (**b**) The distribution of the total amount of science-related grants given or received by philanthropic organizations, compared to grants distributed by NSF and NIH. Note that while the NSF, NIH, and philanthropy all support similar numbers of recipients, there are far more philanthropic donors than recipients.

When we compare philanthropic grants to federal funding, we find that in terms of the amount of funding, philanthropic support for institutions involved in research rivals the funding offered by the top national science funders in the US, with the combined total exceeding the yearly amount awarded by NSF and being comparable to the amount distributed yearly by the NIH (Fig. 2a). Indeed, we find evidence of funds explicitly designated for research or health amounting to nearly $4B/year in recent years (Fig. S3). Note that the increasing trend shown in Fig. 2a exaggerates the true rate of increase in philanthropic support, as the dataset has increasing coverage for the more recent years as more nonprofits filed online, and since as we discuss in SI Sect. II, only 30% to 40% of all funds donated to universities are earmarked specifically for research purposes. Yet, when we limit the data to organizations whose returns are included every year between 2013 and 2018, we continue to observe a 38% increase in philanthropic funding, indicating that foundations did considerably increase their support for science-related institutions in this period.

### The distribution of scientific grants

The non-profit ecosystem varies both in focus and might. We find that across the scientific philanthropic ecosystem, the total dollar amount of grants follows a heavy-tailed distribution (Fig. 2b), indicating that while most organizations distribute relatively small amounts, a few organizations devoted exceptional funds to research. For example, the Gates Foundation distributed over $6.5B in the past 10 years. Overall, the top 200 funders, corresponding to 0.3% of all grantmaking organizations, account for 66% of the total funds given to science. While major foundations dwarf smaller funders in terms of grant numbers and amounts, the long tail of the many smaller funders represents a considerable cumulative impact. For example, more than 7000 funders have each donated at least $1 M over the decade to scientific research institutions, levels of support that could be substantial for many programs.

We find that the distribution of the dollar amount received from philanthropic grants is largely indistinguishable from the distribution characterizing grants from the NSF (Fig. 2b) and that the amount of funds an institution receives from philanthropic sources is highly correlated to the amount they receive from the NSF (Fig. S7). These similarities suggest that the inequality identified in NSF funding is mirrored by philanthropic funding^30,31^. Furthermore, despite the increasing overall amount of philanthropic funding, we find that the distribution of philanthropic funding is highly stable as captured by the stability of the estimated power-law exponent (under 2, evidence of a high level of inequality) and the temporal stability of the Gini coefficient (Fig. S7a). This relatively unchanged inequality since 2010 is in-line with other observations of inequality in science despite the numerous interventions attempted in recent years to mitigate such inequality^32,33^.

### Philanthropy is local

**Figure 3:**
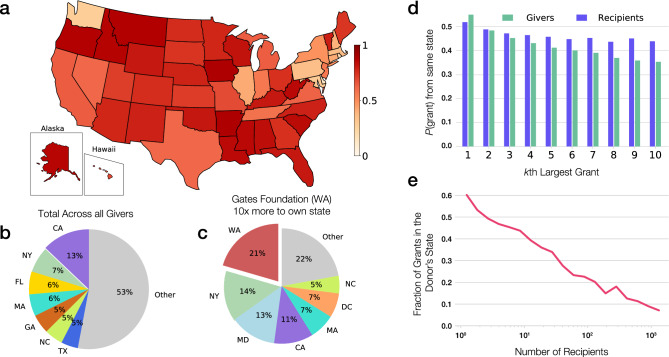
Locality in philanthropy. (**a**) The fraction of dollars given within the state for donors in each state. (**b**) The total cumulative proportion of dollars going to particular states when all donors are considered. Particular grantors are seen to be focused towards their individual states including large funders such as (**c**) the Gates Foundation located in Washington. (**d**) For donors and recipients, we show the likelihood that their *k*th largest recipient was in the same state. For donors we see a decreasing trend, indicating that the largest recipient is more likely to be in the same state than recipients who received less funds. For recipients, while their largest donor is somewhat more likely to come from the same state, the decline for smaller donors is much slower. (**e**) The fraction of grants given within the donor’s home state as a function of the number of recipients supported by the donor. Givers with fewer recipients tend to give more locally compared to those with more recipients.

Despite evidence suggesting federal funding allocation is biased by gender and race^34^, many federal funding agencies have specific mandates to defy geographic boundaries and make awards in accordance with the number and scope of research institutions in a particular location. In contrast, as we show next, philanthropic funding is strongly affected by geography. To quantify the geographic distribution of philanthropic funding, we mapped each non-profit to its state of incorporation and identified grants distributed within the same state. We find that approximately 35% of philanthropic grants go to the donor’s state. On the other hand, if donors were agnostic to geography and distributed grants proportionately across the US such that the number of grant recipients in each state is preserved, corresponding to a degree-preserving random model, only 5% of grants are expected to be awarded in the home state of the donor (Fig. S9a). In other words, the geographic focus of philanthropic funding favors local organizations over 7 times more than expected in the random baseline. We emphasize that the degree-preserving random baseline controls for the fact that scientific recipients are not distributed equally throughout all 50 states as each recipient still receives the same total number of grants (see SI, Section V). We also find that 49% of funds remain within the same state (after removing single-support foundations and donor-advised funds, see SI Sec. IV), compared to 4.5% expected by the degree-preserving random model (Fig. 3a). The difference between the fraction of funds (49%) and the fraction of grants (35%) going in-state suggests that funders not only tend to give more grants locally, but also give larger grants to local recipients. Indeed, we find that over 50% of funders give their largest grant to a recipient in the same state (Fig. 3d). While some foundations are explicit about their focus on local communities, most foundations lack such a mandate, hence, the local focus may be an unintended consequence of their limited ability to engage widely with the scientific community and the scientific community’s failure to reach out to them.

The local focus is reduced for funders who give more grants: organizations with a single beneficiary give 60% of their grants locally, while organizations with more than 1000 recipients only give 10% of their grants locally (Fig. 3e). Interestingly, funders who gave more money did not necessarily give less locally (Fig. S9e,f), suggesting that even those that give significant funds to research may focus their philanthropy on a few local recipients, while disregarding more distant institutions.

Unsurprisingly, large foundations with an explicit local focus and mandate tend to be even more locally focused, with the Lilly Endowment in Indiana distributing over 60% of its funds for science and research in the state of Indiana, the Sorenson Legacy Foundation in Utah giving 83% of its funds in-state and the Dennis Washington Foundation in Montana giving 99% of its funds in-state (see Fig. S8). Yet, this pattern is not limited to organizations with a local mandate. To systematically explore locality for funders explicitly focused on research, we identified 27 large foundations with a declared mission towards advancing scientific research (see SI Section VIII). Often relying on formal calls that defy geographic boundaries, these foundations gave 35,389 grants worth $15.7B over the past decade. Despite their global infrastructure and mission, we find that these major science funders are still locally focused, giving, on average, 30% of their funds to organizations in the same state. For example, the Gates Foundation gives ten times more funds to science institutions within Washington State than expected based on the degree-preserving random model (Fig. 3b,c). Similarly, the Ford and Rockefeller Foundations distributed three times more funds than expected in their home state of New York (Fig. S8). One of the least local funders is the Pennsylvania-based Templeton Foundation, which gives only 1.6 times more to recipients in its own state. In other words, even the largest foundations, who have the infrastructure to seek out national and international applicants, tend to focus locally, either driven by a stated desire to impact their local communities or by unintended network effects, reflecting closer professional and social ties with local researchers and institutions.

At the same time, despite the local focus of philanthropic funders, we find that the level of philanthropic funding to each state largely correlates with federal funding (Fig. S14), suggesting that the geographic funding patterns are tied to institutions’ overall ability to attract funding from all sources and the wealth available in a region. The importance of regional wealth is further supported by two case studies we carried out related to cuts in state funding to higher education. Both Arizona and Louisiana experienced considerable cuts in state funding to higher education, yet Arizona’s economy has grown considerably over the past decade, whereas Louisiana’s has stagnated. In parallel with this, we find that support for Arizona university affiliated nonprofits has grown considerably, whereas support for Louisiana State University’s foundation was flat (SI Section VII, Fig. S21). This suggestive correlation between local economies and scientific support further indicates how philanthropy can be used to create patronage ties and provide legitimacy to funders in their communities.

### Philanthropic funding is stable

**Figure 4:**
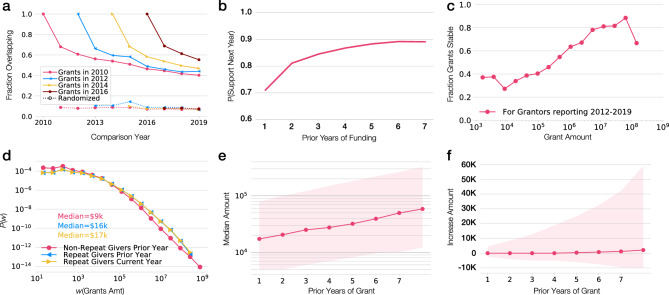
Stability in philanthropy. (**a**) For 2010, we plot the fraction of grants that overlap in future years for the NSF (solid lines) and philanthropy (dotted lines). While NSF funding has greater overlap over shorter periods of 2–3 years, philanthropy has higher longer-term repeated giving. (**b**) The likelihood that a funder will continue supporting a recipient as a function of the number of years of prior support. Grant relationships tend to become increasingly entrenched over time as longer relationships are more likely to continue than shorter ones. (**c**) The fraction of stable grants (continuing for 7 + prior years) versus the grant amount in 2019. The increasing trend suggests that larger donors are more likely to have a stable relationship with their recipients. (**d**) The distribution of grant amounts for grants that do not repeat, the prior year of a repeating grant, and the current year of a repeating grant. The fraction of dollars given within the state for donors in each state. (**e**) The median amount of a grant this year as a function of the number of prior years the grant relationship has existed. (**f**) The median change in grant amount as a function of the number of prior years of the grant relationship.

Another dimension of grant giving relationships is donor retention^35,36^, reflecting the likelihood of continued support given an already established funding relationship. We find that 69% of grant relationships repeat one year later (Fig. 4a) and 60% repeat two years later, compared to 8% repetition predicted in a randomized funding network (Fig. S16a). This high level of donor retention in the foundation space stands in strong contrast to online giving platforms where only 26% of grants repeated one year later^35^. Furthermore, repeated giving becomes increasingly entrenched over time, as donors who gave two years consecutively have an over 80% chance of giving the next year and for the 27,390 funding relationships that have been ongoing for 7 years there is a nearly 90% likelihood to continue in the next year (Fig. 4b).

We find that stable grants (ongoing between 2013 and 2019) are more likely to be given by organizations that offer fewer grants: over half of grantors who give to a single science recipient, support the same recipient every year. The fraction of stable recipients drops to 20% for those giving to a few dozen recipients (Fig. S14b). Similarly, stable grant relationships are more likely to occur when the donor supports the recipient at higher funding levels (Fig. 4c) and are more likely to occur with local relationships (Fig. S14g). Traditional science funders and funds specifically for research also exhibit stability, with 64% of grants repeating in the next year, and their long-term funding relationships also have a 90% likelihood to continue in future years (Figs. S16, S17).

In terms of grant amounts, we find that funders who gave repetitively tend to give more money in the first and subsequent years of their support compared to donors who did not give repeatedly (Fig. 4d). Furthermore, the more years a relationship lasts, the larger the amount (Fig. 4e). At the same time, the typical grant relationship does not tend to involve an increasing donation amount and the median change in funding level after one, two, or even seven years of funding is near zero, indicating that donors give the same amount seven years later as they did in year one (Fig. 4f). This suggests a lock-in effect, such that the initial amount a donor gives to a recipient sets the value of their overall relationship. At the same time, it is worth noting that the variance, particularly to the upside, tends to increase over time, suggesting that while the typical donor does not increase their funding, some donors may offer larger contributions at times. Even still, these increased contributions are typically no more than twice their previous contribution, which is notable given the scale-free distribution of funding, demonstrating that donors’ support remains of approximately the same order of magnitude. One consequence of granting stability is that philanthropic funders of science and higher education may be non-responsive, at least in terms of the resources they are willing to devote, to shifts in the scientific ecosystem and greater need on the part of institutions. The reasons for this warrant future analysis, however one possibility is that the funder has fixed resources available and is already trying to maximize with little room for flexibility to adjust giving as other sources decline.

The stability of philanthropic support is more pronounced over longer time frames than measured for NSF support. Specifically, we find that the NSF tends to maintain 78% of its recipients one year later while philanthropic funders only maintain 68% of recipients one year later (Fig. 4a). However, after two years, the NSF and philanthropic funders maintain almost the same level of recipients at 61% and 60% of recipients. After that point philanthropic funders are more likely to offer repeat or sustained funding to recipients than the NSF. Ultimately, after the 9 years covered in our data, we find that philanthropic funders maintained 40% of their relationships while the NSF only maintained 32%.

Conversely, we find that NSF funding tends to be more stable than 98% of foundations in the ranking of top recipients by dollar amount from year-to-year with a Spearman Rank Correlation of 0.95 (Fig. S17). We hypothesize that this greater stability in federal funding amounts may be the result of an emergent property, where the largest scientific institutions rely on multiple PIs to attract grants, thereby offering greater diversity in potential funding sources. In contrast, while foundations do not systematically increase their support from year-to-year, there is significant variation in funding and many examples exist of one-time major gifts for dedicating new infrastructure or endowments.

### Motifs and clustering in philanthropic support

**Figure 5:**
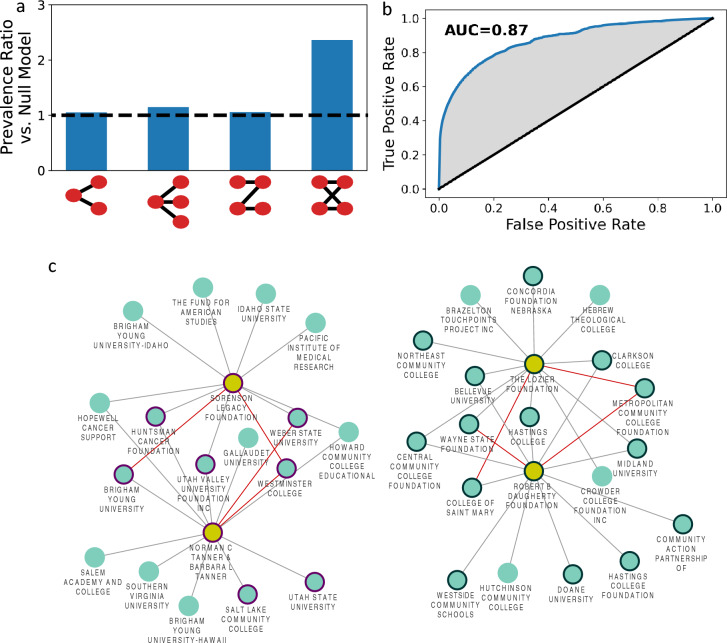
Motifs in philanthropic funding and predictability. (**a**) We show the prevalence of 3-node and 4-node bipartite motifs compared to a bipartite configuration null model. The prevalence ratio versus the null model takes the value 1 when the motif appears the same number of times in the 2019 bipartite funding network and the bipartite configuration model (marked with a dashed line), while a value above 1 means the motif is overrepresented. We see that only the 4-node motif reflecting loops of length 4 is overrepresented. (**b**) The receiver-operator curve (ROC) for predictions using the AA index from 2018 on the network of grants over $10,000 to predict grants over $10,000 in 2019. The area under the curve (AUC) is 0.87. (**c**) Examples of resulting predictions. For four funders (in gold) we show the recipients for whom they were highest ranked (see SI note). On the left we show the resulting prediction network for the Sorenson Legacy Foundation and Tanner Foundation, both located in Utah. We highlight recipients in Utah with a purple border. On the right we show the recipients who ranked the Lozier and Daugherty foundations highest and highlighted with a black border those in Nebraska. We see that the network structure alone identified predictions consistent with the locality of grants. Figure generated with Cytoscape 3.10.

We further analyze the network to understand how its structural properties reflect organizing principles for the distribution of philanthropic funding. Network motifs are substructures where a small number of nodes display a specific pattern of connections between them. Here, we explored the 3 and 4 node motifs present in the 2019 bipartite funding network (see SI Sect. X) and compared the prevalence of these substructures to their prevalence in a bipartite configuration null model. In Fig. 5a, we see that most of the motifs appear at roughly the same rate in the real network as in the null model (within 15%). The only motif that is significantly overrepresented in the observed funding network, by a factor of 2.4 times, is the 4-node motif which captures when two funders both share two recipients (Fig. 5a).

Another way to understand the importance of this 4-node subgraph is through the motif’s relationship to clustering. Interestingly, this motif represents a loop of length four in the network and such loops are strongly related to the bipartite clustering coefficient defined by Robins and Alexander, which examines the ratio of loops of length four to paths of length 3^37^. We find that in the bipartite donation network the value of the clustering coefficient is 0.045, meaning that funders who share one recipient, have a 4.5% chance of sharing another recipient. Notably, this is 135 times higher than expected based on the density of the network, which is just 0.03% (see SI Sect. X). This yet again highlights how similar funders tend to concentrate their support among a shared group of recipients.

While the motif and clustering analysis highlight the prevalence of network structures in which two funders share multiple recipients, our previous analysis on locality of giving suggests that not all pairs of funders are equally likely to have overlapping recipients. To quantify this, we use the pairwise clustering coefficient of Latapy et al.^38^ which asks for two nodes *u* and *v*, how many neighbors they share. We find that that two donors located in the same state are 3.9 times more likely to share a recipient than two donors from different states (2.5% vs. 0.6%) and of all 2,313,046 observed examples of donor-pairs sharing recipients, 15.8% of pairs involve two donors in the same state. Likewise, we find that receivers located in the same state are 2.6 times more likely to share a donor than recipients from different states (5.6% vs. 2.2%). For example, Massachusetts-based Harvard University received funds from 372 distinct foundations in 2019 and MIT received from 284, and 113 of these gave to both institutions, indicating that 40% of MIT donors and 30% of Harvard donors are in common. This strong overlap is not limited to prestigious universities but is a common feature of geographically proximal universities. The University of Nebraska Foundation and Creighton University (in Nebraska) shared 26 foundation donors in 2019, representing 35% of Creighton’s donors and 17% of the University of Nebraska’s donors. Among the notable overlapping donors of the latter institutions, we find several Nebraska-based foundations, such as the Robert Daugherty Foundation which gave $5.4 M to the University of Nebraska and $1 M to Creighton, and the Lozier Foundation which gave $594 k to University of Nebraska and $295 k to Creighton.

While we highlight local overlapping recipients above, many of the four-cycles are the result of some undetected shared common characteristic of the recipients. Indeed, while 34% of four-cycles do include at least two out of four nodes from the same state, only 6.5% of them involve three nodes from the same state and in only 2.3% of cases are all four nodes from the same state. Likewise, if we compare the number of four cycles in the real network to a degree-preserving null model that also preserves proximity, we find 52% fewer four cycles than in the real network. Nonetheless, the null model that preserves both degree and proximity has 23% more four cycles than the null model that solely preserves degree (see SI Sect. X). Thus, while locality can partially explain clustering, additional effects are also at play.

These findings contrast with the common statement used in philanthropy that “if you’ve met one funder, you’ve met one funder”^39^, implying that each philanthropic organization has its own unique and distinct priorities, hence understanding one funder’s approach offers little information on the motivation of other funders. On the contrary, the analysis of network patterns suggests that while each foundation might use its own distinct processes to select recipients, in aggregate, similar funders tend to share recipients. This could be because funders with similar priorities are driven to the same group of recipients, or because overlapping social and professional networks lead funders down the same paths or reflect which institutions can attract philanthropic funds. Regardless of the underlying reason, our results highlight how entrenched funding networks can perpetuate inequalities as institutions not part of the network struggle to find their first funder.

### Predictability in philanthropy

Nonprofits devote considerable resources to find and identify grant opportunities^40^. Recast in the context of our philanthropic funding network, the identification of new funders is a problem of link prediction^41^. We thus examine whether the high levels of clustering in the philanthropic funding network contain predictive power to suggest future funder-recipient pairs. We focus on funders for the 3279 science recipients who received funds from at least five distinct funders in 2018 and remained active in 2019, as well as 17,154 funders who were active in both 2018 and 2019. We used the bipartite Adamic-adar index (AA)^42,43^ to predict the donors that are likely to donate to a specific recipient. This link prediction measure^43^ quantifies the level of structural equivalence between recipients and donors^44^. In other words, if two recipients share some donors, then they are likely to share the remainder of each other’s donors. The metric further assumes that more unique shared recipients or donors convey more information such that, for example, knowing that two universities are funded by a major funder like the Gates Foundation has less predictive value than knowing that they share other more unique funders. Explicitly, the Adamic-Adar index between a donor *s* and recipient *t* is given by,

1 $$A{A}_{s,t}=\sum_{\begin{array}{c}paths\,of\,length\,3 \\ from\,s\,to\,t\end{array}}\frac{1}{{\text{log}}(\left|{k}_{i1}\right|+\left|{k}_{i2}\right|)}.$$

Donor-recipient (s,t) pairs with higher AA_s,t_ scores are more likely to develop a funding relationship than pairs with lower scores (Fig. 5a). We convert the 2018 AA_s,t_ scores to probabilities and test the model’s predictive power by examining whether the predicted grant between a funder and a recipient was awarded in 2019. We find that the predictions obtained from the AA index from 2018 have strong predictive value for 2019, resulting in a remarkably high area under the receiver-operator curve (AUROC) of 0.87 (see SI Sect. X, Fig. 5b). An AUROC score of 0.5 indicates lack of predictive power and a score of one represents perfect predictions. The predictions, as measured by AUROC, remain equally good when we examine funding relationships above a threshold dollar amount ranging from $1 to $10 k, resulting in an AUROC between 0.87 and 0.90. For most research universities the leading prediction tends to be one of a few major funders like the Gates Foundation, Hewlett Foundation, Mellon Foundation, and Charles Koch Foundation, or corporate foundations like those of KPMG, Ernst & Young, or Shell Oil. Given that these foundations fund many universities, for any particular university there is a high likelihood of a grant. In contrast, for community colleges or smaller institutions that have limited access to national funders, the top predictions are often local funders, such as for the Anoka-Ramsey Community College in Minnesota, whose top predicted funder is the Minnesota-based Kopp Family Foundation, which indeed gave over $20 k in 2019.

Next, we inspected cases where the AA index suggests high likelihood of a grant and yet no such grant exists in 2019, finding that these predictions tend to correspond to reasonable recommendations when we consider metadata and a timeframe of multiple years. For example, if we consider the top 100 donors predicted to donate at least $10 k to Harvard in 2019, yet who did not, we find that 76 of them supported Harvard in a year other than 2019. A similar analysis for Creighton University reveals that 22 of the top 100 most-likely predicted donors gave in a year other than 2019. We also find that 18 of the top 100 predicted donors to Creighton who did not give were from the state of Nebraska, suggesting that while these donors may not have supported Creighton, it would be reasonable for them to do so given the previously discussed strong geographical patterns of funding. In Fig. 5c we show examples of two pairs of donors in Nebraska and Utah, along with the recipients ranked highest in their list of most likely funders. We see that several of these recipients previously received from the foundations and that many of the others are also local to the donor organizations. Furthermore, we see that the two Nebraska funders and the two Utah funders share many recipients who ranked them highly while none of the recipients ranked one of the other state’s funders highly.

The observed high predictability^45^ of future donors suggests that there are common patterns in funding outcomes even though each funder has its own unique motivation, focus, governing structure and decision processes. These common patterns and inherent predictability raise equity concerns that funders with similar priorities are highly concentrated towards a few institutions with strong levels of temporal stability. This restricts the ability of other institutions to obtain support and could potentially reduce scientific progress as novel ideas by those at less established institutions go unsupported. However, note that our analysis only focuses on equity across various institutions. If within a single institution there is a diverse pool of research that may benefit from the support, novel ideas may be sufficiently supported.

## Discussion

Our finding that philanthropic support for science is local aligns with other studies documenting the role of physical distance between funders and recipients in philanthropy^46–48^. Yet with 90% of published research papers written collaboratively, and 60% of publications listing authors from multiple institutions and multiple countries^49^, modern science is an increasingly global pursuit that requires access not only to local, but to national and international talent and resources as well. The strong local focus of philanthropy documented above contrasts with these trends. While large philanthropic organizations acquired their funds through national and international investments, most of them are headquartered in already affluent regions, hence their strong local focus can entrench existing geographic inequalities. Indeed, we find that their geographic funding largely mirrors that of other programs, like the NSF, and that changes in philanthropic support in regions may be reflective of general economic trends in those regions, leading to rich-get-richer geographic effects.

The implications of the documented stability in funding patterns are equally multifaceted. Indeed, scientific organizations can greatly benefit from stable funding, offering the opportunity to take risks and focus on difficult problems that require long-term investments. At the same time, stability may also represent inertia rather than an intentional allocation of funds to further specific scientific or research agendas. Furthermore, the stability of support each year highlights the inertial strength of philanthropy, and suggests philanthropy is not responsive to recipient organization needs, such as decreases in state or federal funding for higher education^50,51^.

The predictability of philanthropic relationships suggests that funders may influence one another, flocking to fund similar organizations due to shared priorities. Our predictive algorithm could allow funders to better identify new institutions that they may want to support based on shared interests with other donors. However, this could lead to further concentration of funding among elite institutions. At the same time, it may help focus institutions towards potentially unique research interests, possibly leading to the recruitment of new researchers in those areas. Understanding the full chain of implications of predictability is complex and requires further research both into university behaviors and donor intent.

For individual researchers, the steady increase in philanthropic giving (Fig. 2a) offers increasing opportunities to seek funding beyond the federal funding system^52,53^. Yet, given their familiarity with the federal funding system, researchers tend to limit their fundraising efforts to those large private organizations that have a global presence and regular calls for proposals, operating similarly to federal funders. Our findings suggest, however, that there is exceptional value in engaging with local philanthropic communities, given the strong locality of funding patterns. Such local engagement could enable scientists to directly solicit support from philanthropists, rather than receiving indirect support from general grants that go to the institution’s endowment. Local philanthropy is based more on relationships and outreach, rather than extensive proposals, and can offer more flexibility as philanthropists are not limited to supporting specific programs. Furthermore, the stable funding offered by philanthropists can advance projects with longer time horizons, not yet ripe for national or federal funders. Likewise, the stability of philanthropic funding could serve as an incentive for researchers to increase their scientific outreach efforts as philanthropic supporters of universities have been shown to donate more if they are able to more specifically direct their gifts^54^. Finally, the ability to predict funding relationships enables researchers to better identify and target philanthropists that are more likely to be interested in supporting their institutions, saving time and allowing them to focus their efforts.

Despite the exceptional amount of research and policy focus on national funding, there is limited quantitative understanding of philanthropic giving. Also, most of the existing knowledge relies on interviews and hand-curated datasets^15–17^, with advanced computational methods only beginning to enter the field of philanthropic studies more generally^22^. Here we focused on funding information that can be extracted from US tax forms, offering a foundation for unbiased big-data-driven research to understand philanthropic giving and potentially improve access to philanthropic funds. While the richness of the dataset offered multiple insights, its limitations offer a roadmap for future data collection. First, the tax documents analyzed here are limited to the US, hence the patterns observed here may not hold in other countries. At the same time, there is evidence that philanthropy is playing an increasing role in supporting science in other countries^55^, though in absolute terms the contributions remain modest^56^. Second, these tax documents are limited to philanthropic giving by the approximately 80% of foundations that filed electronically (see SI Sect. I) and do not include giving from foundations who filed on paper or individual contributors. While the existing data somewhat underestimates private support for science, the increasing trends and requirements towards online filing will eventually alleviate this limitation. Furthermore, depending on the versions of the tax form, the employer identification number (EIN), a unique identifier, is not always available. We therefore relied on machine learning (SI. Sect. I) to identify recipients, with potential mismatches for a few organizations due to inconsistencies in the tax forms^57^. In addition, we were only able to disambiguate grants to other non-profits or entities for whom we have an EIN, hence we did not examine grants to non-US organizations and individuals. Finally, not all grants are equally impactful just as not all science is equally significant and some of the recorded grants, while they do support research institutions, only indirectly contribute to scientific research, facilitating instead infrastructure enhancements, undergraduate education, and administrative or programmatic tasks. At the same time, prior work has shown that general funding support does translate into research activity, as measured by publications and patents produced by a university^58^. Despite these and other limitations explored in depth in SI Sec. I, the tax data analyzed here offers the most comprehensive imprint of scientific philanthropy available over the past decade.

To aid the further use of this data for research, we are sharing the cleaned and organized data we extracted from the 990 forms (SI Sect. IX). The resulting dataset is amenable for data mining and other research purposes within the science of science^59,60^. Further efforts are needed to expand this work to track philanthropic funders internationally who may fall under diverse tax laws with different types of reporting. Extensions of this work could help us better understand the nature of the science being funded, linking grants to individual scientists, publications, and patents, allowing researchers to explore the repercussions of locality and stability on scientific productivity and impact, as well as to develop quantitative measures to capture the efficiency of philanthropic and government funding. Importantly, to ultimately capture the full impact of philanthropic funding, future work should explore the specific scientific departments and researchers that benefit from philanthropic funding. Ideally, this should include support that goes to infrastructure (e.g. buildings), endowments, and other indirect support for science. Likewise, such future work should also focus on policy implications, enhancing the relationship between stakeholders, including researchers, policy makers and funders.

More broadly, the approach taken here has important implications for the fields of philanthropic studies and computational social science. Indeed, the area of philanthropy benefits from many government guidelines requiring nonprofits to disclose organizational and financial information. While we focus on the US, transparency rules around nonprofits exist in many regulatory regimes around the world, suggesting our methodology could help map global philanthropic networks. Through the application of novel tools rooted in machine learning, network science, access to systematic philanthropic funding data could improve understanding of the nonprofit space, boost access to and awareness of philanthropic resources, and enable policymakers to increase the impact of nonprofits.

## Materials and methods

### Data collection and filtering

Data was collected from AWS Open 990 filings at https://registry.opendata.aws/irs990/, note that since the end of 2021, the IRS hosts 990 filings directly on its website at: https://www.irs.gov/charities-non-profits/form-990-series-downloads. We identified all grants listed on donors’ tax forms and for cases when only the recipient name and address were given, we applied a string-matching algorithm to determine a unique identifier for the recipient. We then filtered the set of grants down to those that went to organizations involved in science and research including universities and research institutions (see SI Sect. I). The final network in our study consisted of 69,675 donors and recipients and 926,124 grants for reporting years from 2010 to 2019.

### Filtering special cases

Certain donors give large donations that can further bias the appearance of locality. While, across the entire dataset 67% of grant dollars were local, this includes many instances of a university having a separate foundation to receive grants and then making a large grant to the university annually. Therefore, for determining the fraction of grant dollars given locally, we filtered such foundations and other edge cases such as the NCAA, and donor-advised funds resulting in the 49% of dollars donated locally mentioned in the main text. See SI Sec. IV for more on this.

### Bipartite clustering coefficient motif

We explore the motif represented by the bipartite clustering coefficient of Robins and Alexander^37^. This motif,

2 $$C(x)=\frac{4*{C}_{4}}{{L}_{3}},$$

where *C* is the clustering coefficient, *C*_*4*_ is the number of cycles of length four and* L*_*3*_ is the number of paths of length three.

### Supplementary Information

Supplementary Information.

## Data Availability

The final resulting network of science grants is available at https://github.com/Barabasi-Lab/mapping-philanthropy/. See SI Sect. IX.
