# Supplementary material for: Mapping philanthropic support of science
Source: Sci Rep. 2024 Apr 24;14:9397. doi: 10.1038/s41598-024-58367-2 (PMC11043411; doi:10.1038/s41598-024-58367-2)
Supplement: Supplementary file 1 — Supplementary Information. [file 41598_2024_58367_MOESM1_ESM.pdf]

Mapping Philanthropic Support of Science

Supplemental Information

Louis M. Shekhtman[1], Alexander J. Gates[1], Albert-László Barabási[1,2,3,4]

[1]Network Science Institute, Northeastern University, Boston, Massachusetts 02115, USA

[2]Center for Cancer Systems Biology, Dana-Farber Cancer Institute, Boston, Massachusetts 02115, USA

[3]Department of Medicine, Brigham and Women's Hospital, Harvard Medical School, Boston, Massachusetts 02115, USA

[4]Department of Network and Data Science, Central European University, Budapest 1051, Hungary

CONTENTS

I. Data Collection and Processing 3

II. Examining purpose statements 8

III. Separating Givers and Receivers 10

IV. Network Map of Philanthropic Funding 11

V. Filtering Exceptions: Single-Support Foundations, Donor Advised Funds, & Sports Conferences 15

VI. Understanding the Locality of Scientific Funding 18

VII. Regional Characteristics and Science Philanthropy 21

VIII. Understanding the Stability of Scientific Funding 23

IX. Analyzing Traditional Science Funders 26

X. Donor Overlap of Recipients and Link Prediction 26

XI. Data Availability 29

XI. Data Availability 29

I. Data Collection and Processing

Initial Data. We collected the IRS 990 filings from https://registry.opendata.aws/irs990/. In total we downloaded 3,910,398 tax forms for 685,397 organizations, of which 3,660,949 were filings for tax years from 2010-2019. While 2,281,346 (62%) of the included tax forms were filed for a period ending Dec. 31st, another 1,379,603 (38%) filings did not use a standard calendar year reporting period. For these filings, the year of the filing period end date was used to assign the grants to a particular year.

Two important data limitations should be noted. First, our data only includes tax forms for the organizations that filed electronically; paper tax filings are not contained in the database. The growth in the number of organizations filing online over the previous decade contributes to the trend of increasing philanthropic support (main text Fig 1a). To evaluate the extent of organizations potentially missed by our e-file restriction, we compared the 431,896 non-profit organizations covered in our complete dataset in 2016, to an estimate from the National Center for Charitable Statistics of 533,112 organizations required to file a form 990 or 990-PF for private foundations (see https://nccs.urban.org/publication/nonprofit-sector-brief-2019#number), concluding that we cover 81% of all non-profits in that year. A similar calculation using the 2019 IRS Business Master File suggests that 582,436 organizations were required to file Form 990/990EZ or 990 PF and our dataset contains returns for 460,937 organizations (79%). Thus, we are confident that we capture the finical records for a strong majority of all non-profit organizations in the US. Second, the IRS tax forms only include grants given by a non-profit organization, and do not include contributions given directly by individuals.

Disambiguating Organizations. We focus on the over 10 million grants disclosed on the givers' tax forms (see Fig. S1). In 35% of the grants (3,678,608) the recipient's Employer ID Number (EIN) was listed, uniquely identifying the grant recipient. In the 6,710,171 remaining grants, only a name and address of the recipient was provided. We then excluded 224,949 grants made to recipients outside of the US. To identify the EINs of these recipients based on address, we used the IRS business master files (BMF) containing a complete listing of basic information of all non-profit organizations, including the address and EIN. In addition, some grants listed a recipient name, which was not the official legal name (e.g., 'Harvard University' rather than the legal name 'President & Fellows of Harvard College'). To overcome this issue, we first created a listing of all non-profit organizations listed in each state in the BMF and compared the recipient name listed by the foundation to all of the organizations registered in that state. We used the TFIDFVectorizer in the sklearn Python package to establish word-based substrings of a name that are most identifiable and then word tokenized the listed name and the possible matches in that state. We assigned a preliminary match based on the possible match with the maximum cosine similarity between the tokenized word sets. Matches that had a cosine similarity above 0.75 were preserved. Likewise, matches that had a cosine similarity above 0.5 and also had a zip code that was identical to the zip code listed by the foundation were preserved. This procedure allowed us to uniquely identify the EIN for 4,507,447 additional organizations.

In total, we disambiguated the recipient for 8,186,055 grants of 10,388,779 total grants from 2010-2019. To assess the accuracy of our matching, we examined by hand the grants from the Gates Foundation in 2019—a total of 2,393 (including multiple grants to the same institution). We successfully matched 2,038 of these. 1,800 of these matches had the exact

name listed on the Gates Foundation return as the entity we identified. In total, we found 54 matches that appeared to be incorrect (although five of these represented donations to foundations affiliated with the correct entity e.g., the Georgia Chamber of Commerce as opposed to the Georgia Chamber of Commerce Foundation). Likewise, many of the errors involved matching to private companies or public institutions with similar names to non-profits e.g., a grant to a city as opposed to a non-profit with the city's name in it. We further reviewed the institutions we failed to match which included many for-profit companies in the medical space, and public entities like schools and cities. Overall, we believe that similar issues occasionally precluded our ability to successfully match the remaining grant recipients for other givers. It also must be mentioned that some foundations may have listed non-standard names for a recipient to the point where our algorithm was unable to match them.

To further assess the accuracy of our matching algorithm we randomly sampled 400 grants from our network in 2018 and hand-checked the matches. We found that only 1.5% appeared to be mis-matched as not going to either the intended institution or an affiliated institution. If we further examine only the grants that required matching, we find that 215/221 (97.2%) were correctly matched to either the institution or an affiliate of the institution, only a slightly higher error rate than for the Gates Foundation.

Filtering to Science. After reducing our dataset to those grants where a recipient EIN was successfully determined or provided, we then filtered these grants down to organizations involved in science. The IRS uses the National Taxonomy of Exempt Entities (NTEE) for classifying non-profit organizations (https://www.irs.gov/pub/irs-tege/p4838.pdf). There are 26 main codes, each corresponding to a letter of the alphabet. We defined scientific non-profit organizations to be organizations classified under one of three general categories: Medical

Research (Codes beginning with 'H'), Science and Technology Research Institutes (Codes beginning with 'U'), and Social Science Research Institutes (Codes beginning with 'V'). Additionally, we included organizations classified within one of two subcategories of the Education category (Codes beginning with 'B'), specifically: Higher Education Institutions (Codes beginning 'B4') and Graduate Schools (Codes beginning 'B5'). The NTEE codes for non-profit organizations were obtained from the BMF. We included organizations classified under one of our science categories in either the 'NTEE_CD' or 'NTEECC' column in the BMF.

Since our aim is to comprehensively capture scientific research, we also account for public universities that are not non-profits (and thus not in the BMF) and university-affiliated foundations assigned to a different NTEE code. We identified 3,738 EINs that were listed as having received a grant and whose name suggested they were involved in higher education. In addition to public universities, many of these organizations were classified under 'B11' for Single Organization Support i.e., a nonprofit organization set up to collect funds on behalf of another organization which is typically a public university in our case. We compared our list to the Carnegie Classifications of Higher Education (https://carnegieclassifications.iu.edu/downloads.php) and determined that all 166 public doctoral/research universities were represented in our data and that of the 1665 public-controlled institutions in the Carnegie Classifications 1296 (78%) had a perfect match among our added institutions using a TFIDFVectorizer with word-based substrings. We reviewed the remaining public institutions listed in the Carnegie Classifications and noted that in many cases these involved multiple campuses within the same university system (which from an organizational perspective may not have separate EINs) and small community colleges that likely did not receive any or many grants from philanthropic sources.

Overall, we find that the majority of research grants go to universities (Fig. S1), followed by medical research institutes, science research institutes and a smaller number going to social science research institutes. We find that 92 of the top 100 recipients by grant amount are universities with the remaining 8 made up of medical research institutes and a climate research institute.

It is worth noting that we are only able to assess grants given to the primary institution, and are unable to disambiguate grants to individual researchers, scholarships, or 'pass-through' grants. This means that if a grant is intended for an individual or as a subaward for another institution, it will be attributed to the primary institution. In contrast if the grant recipient listed is an individual, we will be unable to disambiguate the recipient since the individual will not have an employer identification number (EIN) and thus will not be in our final corpus.

Federal Funding Collection. Data for NSF was collected from the NSF website on Dec. 18th, 2020. The NIH data was collected from the bibliometric database dimensions.ai. We include all recipient organizations of NSF or NIH grants, including universities, research institutes, and for-profit companies.

Most NSF and NIH grants only disclose the total amount of funding for the life of the grant and the years in which the grant was active. To estimate the yearly amount of these grants, we divided the total funding amount by the number of years for which the grant was active. Furthermore, when counting annual numbers of grants, this process will likely increase the estimated number of grants each year since we count the grant for any year it is active e.g., a 3-year grant from June 2018-June 2021 will be counted as active for 4 years from 2018-2021. The NIH reports the number of grants based on their fiscal year

(https://report.nih.gov/funding/nih-budget-and-spending-data-past-fiscal-years/budget-and-spending), which ends at the end of September. Totaling their reported grants across all categories leads to an estimate of e.g., around 56k grants in 2019, less than our estimate of around 81k grants, likely due to our counting of grants for any calendar year they are active as opposed to according to the NIH fiscal year. However, this bias only reinforces the increasing importance of philanthropic funding as our estimate of the number of federal grants is an upper bound.

II. Examining purpose statements

The IRS filings listing grants contain a stated purpose for the grant which is a freetext column provided by the donor. We examined the most common purposes and hand-labeled the top 200 purposes classifying them into one of the following categories: research, general support, education, subawards, scholarships, health, athletics, religion, broadcasting, single support, donor advised funds, and NCAA sports conferences. In cases where a grant mentioned two categories e.g., Research and Education, we split the grant with half of the funds going for research and half going for education. Aside from our hand classifications we further classified any grant from a single support foundation as being single support regardless of the purpose (often times these grants list that the associated foundation is funding research or student scholarships), any grant from a donor advised fund as being from a donor advised fund, and any fund from a sports conference or the NCAA as having come from the NCAA or a conference.

In addition, we used the following string matchings to assign purposes across many grants. First, any grant that contained the words ‘subaward’, ‘subcontract’, ‘subgrant’ or ‘sub-recipient’ was classified as being a subaward. Any grant whose purpose mentioned ‘program support’ ‘general support’, ‘unrestricted’, ‘further mission of organization’, ‘exempt purpose’,

'charitable' or 'charity' was classified as being general support. Any grant that mentioned 'educational assistance', 'educational activities', 'educational institutions', 'for education' was classified under education. Any grant that mentioned 'health' was classified under health. Any grant that mentioned 'athletic', 'athlete', 'ncaa', or 'sports' was classified under athletics. Any grant that mentioned 'research', and did not mention the grant being for a subaward and was not from a single-support foundation was classified as research.

In Fig. S3a we show the total amount of grants categorized under each of the twelve categories, indicating that the bulk of non-profit support for universities and science institutions falls under general support and support from single-support organizations (like the university's fundraising foundation or affiliated hospitals).

Overall, previous estimates[14] suggested that for the largest universities around 40% of their philanthropic support was directed towards research-related activities. This is comparable to the 31% of funds that we find for Research or Health purposes after excluding grants whose purpose is not defined such as Single-Support grants, grants from the NCAA, subawards, grants from donor-advised funds, and grants that were not classified. The gap between the reported 40% and the observed 31% may also be due to the fact that some of our grants listed in 'Other' category may be research related. Furthermore, the previous work also included endowment funds for research, which are often listed as 'General support' by the funder and may be responsible for a significant portion of the difference in the estimates.

We find that funds classified for research or health purposes tend to be slightly less local than grants for general purposes, education, or scholarship yet are still locally focused with 35% of dollars and 24% of grants remaining in-state (Fig. S3b). We further find that when

we consider the set of grants whose purposes lead us to classify them as research or health, or that came from a traditional science funder (see Sec. IX), that our results remain robust in terms of both donor retention and stability, and locality (Fig. S17)

III. Separating Givers and Receivers

To understand the different roles of organizations in our network, we classified the 69,675 organizations in our network based on the grants given and received. We found a clear separation between givers and receivers. 56,144 organizations (80.6%) only gave grants to scientific organizations, 11,151 organizations (16.0%) only received grants in science, and the remaining 2380 organizations (3.4%) both gave and received grants. Overall, we find that a binary classification of givers or receivers appropriately classifies around 97% of organizations in our network.

The remaining 3% of organizations that both give and receive funding in science is largely composed of three main types. (i) First are, major universities, who are primarily recipients but occasionally redistribute funds to other universities or entities acting as subcontractors or collaborators. It is worth noting that even though many grants involve subcontracts, there are relatively few organizations which have the administrative capacity to coordinate the subcontracts. (ii) The second type of organization involved in both giving and receiving are fundraising organizations affiliated with universities or other institutions that raise funds exclusively for a single institution e.g., the Gothic Corporation, a separate nonprofit formed by Duke University to fundraise on its behalf or foundations for public universities like the University of Illinois Foundation and University of Wisconsin Foundation. (iii) Finally, medical research foundations and other professional scientific foundations may both solicit

donations and distribute funds for research e.g., the Michael J. Fox Foundation for Parkinson's Research or the American Physical Society.

In Fig. S4, we plot the number and amount of grants given and received for the subset of organizations that both give and receive grants. In the case that a binary classification is necessary for all organizations, we use the number of grants as a determinate of whether an organization is a giver or receiver since this will accurately classify single-support organizations and most of the universities that both gave and received grants. Since 97% of organizations can be classified as solely being either givers or receivers in the science space; the classification of the few remaining organizations should not significantly affect the qualitative conclusions we draw.

IV. Network Map of Philanthropic Funding

To build a complete map of philanthropic funding in science, we aggregated across all grants from 2010-2019. We note that the total number of grants can include multiple grants from a single grantor to the same institution in a single year. For example, the Gates Foundation gave 2 grants to Princeton University in 2018. In many cases, these grants represent funds for distinct purposes, yet at times they may only represent separate payments for the same purpose. Since the differences depend on each funder, we chose to count each listed grant separately. The total amount from a giver to a receiver reflects the sum across all grants over the entire decade.

The largest givers by number of grants tend to be 'donor-advised funds.' These funds are operated by banks on behalf of their clients. This structure allows the giver to claim the tax-break in the year of the donation and only later determine the recipient. Nonetheless, in the database an entire donor advised fund will appear as only a single entity, even though it

reflects the giving of many smaller donors directing these accounts. While clients specify where to donate their money, the reported grants reflect aggregates over all clients. The largest donor-advised funds (by number of grants) are those operated by Bank of America, Charles Schwab, Fidelity, and J.P. Morgan Chase. Other major givers include the NCAA (which redistributes broadcasting income and other income sources), large private foundations like the Gates Foundation, corporate foundations such as the Shell Oil Company Foundation and the Ernst & Young Foundation, and community foundations such as those in Columbus, and Houston. In Fig. S6a we show some of the top givers. Other large private foundations by number of grants are the Templeton Foundation, Moore Foundation, and Robert Wood Johnson Foundation.

The largest grant giver by amount is the Gates Foundation. Many other top givers by amount are single-support foundations that fundraise on behalf of a single university or institution, such as the Gothic Corporation for Duke University and the Georgia Tech Research Corporation, as well as foundations affiliated with major public universities. Also appearing on the top lists are the Big Ten Conference, several university affiliated hospitals that transfer funds to their parent university, and even a few universities themselves who presumably were involved with redistributing funds. In Fig. S6b, we excluded these examples and found that the largest givers by amount after the Gates Foundation were the Hewlett foundation, the Corporation for Public Broadcasting and the American Heart Association, who each gave over $1B over the decade. Other top givers by amount included the Lilly Endowment, Moore Foundation, and American Cancer Society.

The top grant receivers by number (Fig. S6c), tend to be major universities, including UPenn, Stanford, Michigan, Yale and Duke. The top non-university recipients were the Dana

Farber Cancer Institute and Memorial Sloan Kettering. The listing of top recipients by amount (Fig. S6d) can be strongly biased by single-support foundations as these can give very large amounts to a university. However, this is often not truly representative as these foundations accumulate numerous funds on behalf of the university, including from private individuals. In contrast, grants given directly by individuals to other institutions without a separate single-support foundation are not found in our data. Therefore, we also include the list of top recipients in Fig. S15 which excludes support foundations, affiliated hospitals, and donor-advised funds.

When we examine the distribution of amount received in philanthropic funding at an institution level, we find that it is largely identical from year to-year (Fig. S7a) following a long-tailed distribution. If we estimate a power-law exponent for this distribution, we again find it to be largely similar from year to year and considerably below 2 (Fig. S7b). A similar stability is observed in the Gini Coefficient (Fig. S7c) from year-to-year. This stability highlights the persistent levels of inequality in philanthropic funding over the period of our study and follows with other work on the topic.[25,30,31] Furthermore, when we limit our work to only include grants that are related to research (either based on purpose statements, or because they come from a known science funder), we observe similar patterns (Fig. S7d-f).

In the main text, we also compared the distribution of philanthropic funding and federal funding. In Fig. S6e-f we show a different way of visualizing the difference by plotting the fraction of total grants and funds from/to the top 200 organizations, the next 201st-2000th organizations, and the remaining organizations. We find that over half (51%) of philanthropic grants are given by organizations outside the top 2000 givers, suggesting that a broad base of funders supports science. Likewise, the top 200 recipients by number of grants received only

around a third (37%) of philanthropic grants, whereas top recipients of federal grants received 76% and 79% of NIH and NSF grants respectively, again demonstrating the greater breadth of philanthropic support. Nonetheless, when examining the total funds received by different groups of ranked institutions, we find that these are more similar with the top 200 recipients attracting 73% of philanthropic funding, 83% of NSF funding and 81% of NIH funding. This suggests that while more organizations may be able to compete for philanthropic funding, there remain a limited number of institutions that receive most of the funds.

There are strong relationships between the number and amount of NSF and philanthropic funding given to recipient organizations. We first matched organization names between the NSF and philanthropic recipients using the TFIDFVectorizer with word-based substrings as was done earlier to fill in recipient EINs. We reduced the matches to those with a cosine similarity above 0.8 leaving us with 2878 matched organizations between the NSF data and philanthropic data. In Fig. S7, we show that there is a clear relationship in both the number and amount of grants received from philanthropic sources and NSF (Pearson and Spearman Correlations of >0.45 in all cases and p-values <1e-10 in all cases, see Fig. S7a-b). However, despite this strong correlation there remains considerable spread in the level of federal funding even for a given level of philanthropic funding (Fig. S7c).

We further identified 2133 philanthropic organizations that also received from both the NSF and NIH. These 2133 philanthropic recipients represent only 16% of all receivers, yet they received 51.5% of all philanthropic funds, highlighting that most major institutions tend to receive from all three funding sources. Nonetheless, there remain many other smaller recipients that only received from one of these sources.

V. Filtering Exceptions: Single-Support Foundations, Donor Advised Funds, & Sports Conferences

The US non-profit ecosystem contains many special cases including 'single-support foundations' set up by an institution to fundraise on its behalf; donor advised funds, usually operated by a bank, who collect funds on behalf of many individuals and disperses the funds to charities on their behalf and at their direction; and sports conferences like the NCAA, Big Ten, Big Twelve and others. Such unique cases can significantly affect some of the results, since these nonprofits tend to control significant amounts of funds. Here we discuss which analyses are most affected by these special cases.

First, are single-support foundations that tend to give annual large grants to the parent institution through funds they've collected from other donors, including private individuals. These foundations can thus skew the appearance of philanthropic funding as not all institutions form such foundations and the large grants from them may artificially increase the appearance of philanthropic support. To identify these foundations, we filtered nonprofits that received multiple grants and then only gave a single grant. Such institutions are likely to be single-support foundations that fundraised from various sources and then donated the funds to the parent organization, identifying 462 such foundations. We supplemented this list with an additional 42 manually identified single-support foundations or university hospital affiliates. Aside from this list, we also included all institutions classified under the NTEE Codes of 'B11', 'U11', 'H11' and 'V11' which refer to single support foundations, as well as any donor classified under 'E' for healthcare, which is often likely to be a university affiliated hospital. We note that the NTEE classifications are not perfect and thus we may filter some foundations that are not

affiliated with the recipient institution, however the majority of those filtered are university affiliates. Overall, we identify 4,914 organizations that may act as single-support organizations. Single-support foundations will especially bias analyses related to grant amounts, since these organizations give single large grants. This will lead greater amounts of funds to appear local since the single-support foundation is generally co-located with the parent organization. Second, the single-support foundations will somewhat increase the appeared stability of grants. However, because single-support foundations represent a relatively small fraction of grants (since each single-support foundation only gives one grant generally), the bias they cause in this respect is fairly small.

Second, are donor-advised funds (DAF) and sports conferences like the NCAA. Since DAFs combine many individual donors directing funds independently into a single foundation, they will obscure certain aspects of our analysis. First, DAFs will appear less local than other types of foundations because funds from donors throughout the country are combined into a single foundation. Therefore, even if a donor in one state gives to a local institution, the grant may not actually appear local since the donor advised fund is based elsewhere, prompting us to understate the locality bias in giving. Similarly, DAFs will overestimate stability as even if a different donor donated to a particular recipient institution each year, the DAF will have a grant to them and the grant will appear stable. Again, while DAFs have many recipients, they still represent a small fraction of total grants and thus the bias in this respect is fairly small. For the analysis in Fig. S15, which replicates the key aspects of the main text, we removed eight DAFs, corresponding to Fidelity, Charles Schwab, the National Philanthropic Trust, Raymond James Charitable, the Jewish Communal Fund, Goldman Sachs Philanthropy, Bank of America and the National Christian Foundation.

Sports conferences like the NCAA, Big Ten, Big Twelve, etc. will bias the results in a manner similar to DAFs as proceeds from advertising doing to the different institutions will be aggregated and then divided. Thus, these organizations will often appear less local (since the grant recipients are throughout the region of the conference or country), and more stable (though again they have comparatively few grants). For the analysis in Fig. S15 we removed major collectors of sports revenue including the NCAA, Big Ten Conference, PAC-10 Conference, Big Twelve Conference, and Big East Conference.

The key impact of the removed donors is reflected in changes to analyses that focus on the amount of grants, especially the amount of grants given locally. In Sec. V below, we show that across all donors 67% of dollars are given locally, however after we remove single-support foundations, DAFs and sports conferences, this is reduced to 49%. Even at 49%, the fraction of dollars given locally is significant and is well-above the fraction of grants given locally, reaffirming the point that donors tend to give their largest grants locally. In the main text, we chose to cite this 49% figure as opposed to the 67% for all donors since it better reflects the locality of typical philanthropic donors. After excluding the aforementioned exceptions, the fraction of grants given locally increases slightly to 36%, however because the numbers are so similar in the main text we referenced the 35% of grants local which includes all donors. Similarly, our rankings of top recipients by amount of funds also change significantly, though the top recipients by number of grants remain similar with just a few organizations shifting positions (see Fig. S15).

VI. Understanding the Locality of Scientific Funding

To explore the relationship between locality and philanthropic funding, we define locality at the state level, which is easily determined for all organizations from the IRS BMF (see Section I, above). We can illustrate the diversity of local giving through a few case studies as shown in Fig. S9. The geographic distribution of funding for several large funders, including well-known foundations such as the Gates Foundation and Rockefeller Foundation, as well as somewhat lesser known, but still large foundations such as the Lilly Endowment, the Sorenson Legacy Foundation, and the Dennis & Phyllis Washington Foundation. We see that most of these foundations have a strong bias towards giving within their own state. Even the Gates Foundation, which makes clear efforts towards emphasizing intellectual merit and broader impacts, still has a strong, detectable bias towards Washington State. For the Rockefeller foundation, more funds are sent to Washington D.C. than remained in New York, though New York still receives over 20% of the funds. Meanwhile, the foundations not known to solicit proposals from scientists, such as Sorenson, Washington and the Susan Buffett Foundation have an explicit local focus and give a majority of their funds within their own state.

In Fig. S9a we find that around 35% of grants are between givers and recipients located within the same state, representing 67% of funds by dollar amount. We then carried out 100 simulations of a random null model of grant giving in which grants are shuffled randomly in a degree preserving randomization. The degree preserving null-model can be thought of as taking two links (grants) from two funders to two recipients and swapping the ends so that the links from node A to B and node C to D become links from A to D and B to C. If this process is done iteratively many times, the overall degrees of each node are preserved, yet the structure of the network is randomized. In our case, this means that we are preserving the size of each

giver and recipient i.e., the Gates Foundation still gives many grants and Harvard still receives many grants, however the patterns of giving are altered. This enables us to compare the actual network to the randomized version. The measured number of locally given grants is 7x greater than would be expected compared to the null model and the amount of locally given grants is 13x more than would be expected in the null model.

We find differences in local giving related to the number of grants given by the grantors. In Fig. S9b-c, we show that grantors giving grants to more institutions, i.e. having higher out-degree (k_{out}), tend to give more of their grants and dollars outside of their own state. These differences are particularly pronounced in comparison to receivers, where those receiving more grants continue to receive a large fraction of their grants and dollars from within their state (note that Fig. 3e from the main text is identical to Fig. S9c for k_{out}). The total amount of funding (w_{out}), shown in Fig. S9e-f, displays a much less pronounced effect, with some of the largest grantors giving a larger fraction of funds within their own states. We suggest that this can be explained by noting that funding more research institutions requires greater effort on the part of the foundation and thus suggests an overall greater interest and commitment to identifying meritorious research directions. Conversely, the total amount given by a foundation is related more with the wealth of that foundation and does not necessarily imply that the foundation has a keen interest in scientific research. Thus, even wealthy foundations may donate a few very large grants towards local institutions that they identify with rather than seeking out research institutions farther away.

In Fig. S10a-b we show differences in the fraction of grants and dollars given in-state by funders from different states. We note that there are large deviations across the different states and few clear patterns exist. However, comparing the two choropleths suggests that the

fraction of dollars given in-state tends to surpass the fraction of grants given in-state, reiterating our finding that the largest grants tend to be local and that even if funders seek out distant recipients they tend to provide the largest portion of their funds locally. When we normalize the fraction given in-state by the fraction that state received from all funders, we find that smaller states all tend to have a more significant bias towards their own state- over 10 times greater giving to their own state compared to what their state receives overall (Fig. S10c-d). At the same time, while the bias of larger states is smaller in magnitude, it reflects a considerably greater proportion of funds. Comparing in-state receiving vs. in-state giving in Fig. S10e-f, we find considerable differences across the states, though again few discernable patterns emerge.

In Fig. S11, we show the locality of giving by county. We first matched city-state pairs to counties using Google's geocoder, obtaining county information (FIPS codes) for 68,998 organizations in our sample (99%). We then examined locality at a county level finding that 25% of funds and 15% of grants were given within the same county as the donor. This was far greater than the average of 20 realizations of a degree-preserving null model where only 1% of funds and grants were local at the county level.

We can further compare differences in giving between the coasts. An important question is whether the locality is only because of the concentration of top institutions in major coastal states (NY, CA, etc.). We can address this by examining the giving from an east-coast state to a west-coast state to see if there is increased giving between them. To do this, we constructed a degree-preserving null model where each giver shuffles the recipient of each grant. Over 100 realizations of the null model, we find that on average 11.9% of funds from New York donors went to California recipients, yet in the real-data only 7.4% of funds from

New York went to CA, suggesting not only is there not increased giving between these regions, but rather there might be decreased giving, though this is not significant (p=0.19). In addition, in the null model 10.4% of funds from California donors went to New York recipients, yet in the real data only 3.0% of funds from California donors went to New York recipients, with the reduced funding from California to New York even being statistically significant (p<0.01). Overall, these results suggest that locality does not appear to be merely the result of a concentration of major institutions, but rather that donors focus locally to the exclusion of other more distant major institutions.

VII. Regional Characteristics and Science Philanthropy

A key question inspired by the strong locality in philanthropic funding of scientific institutions in the US is whether this affects the overall geographic patterns of funding. In Fig. S12 we compare the number and amounts of NSF and Philanthropic funding across states. We find that both the number and amount of funding from philanthropic sources is highly correlated with NSF Funding; the number of grants has a Spearman Rank Correlation of 0.85 and the amount of grants has a Spearman Rank Correlation of 0.94. Thus, while individual funders are strongly biased locally, the overall geographic pattern of funding is similar between government and philanthropic sources.

Another question arising from the regional analysis is whether we can differentiate between different 'types' of wealth, such as 'old money' vs 'new money'. Examining this question using the tax data is difficult since the source of a foundation's money is not easy to discern and benefactors may close and open foundations at their will. Nonetheless, the IRS shares the year in which an organization is ruled as a non-profit in the Business Master File. We identified the ruling year for 12,984 of our funders. In this subset, the oldest listed ruling year is 1900 and

the most recent are from 2020. Overall, the median ruling year is 2001 with only 10% of organizations having a ruling year prior to 1966. If we examine the median ruling year for donor organizations in different states weighted by their amount given, we see that funding from the Northeast tends to come from earlier ruling years (Fig. S13) suggesting that perhaps Northeastern donors to scientific institutions are more likely to come from 'old money.' At the same time, few donors are more than 50 years old, which suggests that the majority of foundations are still not particularly old. We note also that older organizations tend to give somewhat more money (Spearman rank=0.13, $p<1e-50$).

A further aspect of regional considerations is how public universities might be able to turn to philanthropy for support in cases of cuts to higher education. To begin exploring this question, we examined a few institutions in states that reported significant cuts in higher education. Specifically, we looked at Louisiana and Arizona, two states with some of the biggest cuts to higher education since 2008.[47-48] We examined the University of Arizona Foundation, the ASU Foundation, and the Louisiana State University Foundation. We find very little evidence that philanthropy has changed in response to funding cuts: while for ASU and the University of Arizona the amount of foundation giving does increase, for LSU funding remains basically flat from 2014 to 2019 at around $2.4M (Fig. S21). This highlights how there are likely to be regional differences in the public institutions that have access to philanthropic resources to possibly mitigate cuts to higher education. Furthermore, we see that supporters of public universities in both Arizona and Louisiana did not systematically increase their support over time (Fig. S21c-d), in line with how most funders were not seen to systematically increase their support (Fig. 4f). Despite these preliminary findings, further work is needed to

disentangle the effects of state policies, regional wealth, and philanthropic funding of higher education.

VIII. Understanding the Stability of Scientific Funding

To explore the stability of scientific funding, we expand our map of philanthropy to a temporal network built in yearly snapshots from 2010 - 2019. We find that nearly 68% of edges are preserved between two consecutive years, and that around 60% repeat 3 years later (Fig. 4a). This is significantly greater than would be expected in a null model where each year's grants are shuffled such that each giver (receiver) has the same number of grants given (received) in each year. Across 100 samples of these random networks, we find that, on average, only around 4% of grants are preserved across two consecutive years.

We next examined 'stable grants' namely those that repeat every year from 2013-2019. We find that stable grants are more likely to come from grantors who give out fewer grants. To show this, we first identify the set of grant-givers whose IRS forms are contained in our dataset for all years from 2013-2019. We then examine the grants given by these givers to science in the year 2019. If that grant was also given every year from 2013-2019, we say that is a stable grant. We find that 80% of grant givers who give out only a single grant to a scientific organization, give to the same recipient every year (Fig. S14a). This fraction decreases to around 20% for those giving out around 20 grants, and then trends upward as k_{out} further increases. However, this later increase in stability is most likely related to the limited number of large research institutions as the null model shows a similar increase for high values of k_{out}. A particular concern with this analysis is the possibility that single-support foundations could bias donors who give only one grant. We therefore dug deeper into the 2,079 givers who only

had a single science recipient in 2019 and who filed a tax form every year. Among this subset we find 1487 foundations who also supported at least one organization with an IRS classification outside of our science set. Presumably these givers are less likely to be single-support foundations since they gave to other institutions outside of science. Nonetheless, we find that 1085 of organizations in this subset maintained a stable science recipient. Furthermore, the median grant amount from these 1085 organizations was only $15k, an amount far below what one would expect an affiliated foundation to be giving to a university. Similarly, if we limit ourselves to those foundations that gave less than $1M in 2019 (a low threshold of what a support foundation for a small college could be expected to raise), we find that there are 1425 funders remaining and that 1029 (72%) of these maintained a stable recipient, which suggests that donors who give only a single grant to science are indeed more likely to pick a single recipient e.g., their alma mater.

Grant relationships that involve larger dollar amounts are more likely to be stable (repeating each year from 2013-2019) than grants of smaller amounts (main text Fig. 4c). At the same time, for grant amounts above $10M caution should be taken in making broad interpretations since there are only 206 such grants. Moreover, many large grants, especially those >$100M are between related organizations such as a support foundation giving to its parent organization and the stability of such relationships is not surprising. Nonetheless, the increasing trend from around $10k to $1M indicates that many givers who give in greater amounts do give more consistently. Similarly, grants that continue across multiple years are likely to involve higher annual amounts (main text Fig. 4e). To better understand this, we measured, across particular donor-recipient pairs, the median increase in grant amount following *n* years of prior support. We find that the amounts do not tend to increase

significantly over time (Fig. 4f), with the median increase one, two and three years later being 0. After 7 years, the median increase is $1185 though such grants were for a median of $50k (main text Fig. 4f) meaning that the increase was barely 2%. This suggests that those grants that continue for a longer time tend to start out with a higher level of giving rather than growing over time. In other words, while donors tend to remain loyal to specific research institutions over time, they don't tend to increase their giving much if at all. Lastly, stable grants are somewhat more likely be local than non-stable grants (Fig. S14b).

In the main manuscript, we further compared the stability of philanthropic funding to NSF funding (Fig. 4a), finding that while over shorter periods of 2-3 years NSF funding was more stable, over longer periods philanthropic funding had higher rates of repeating. In Fig. S17 we further compare this stability by examining the rank stability of the total amount given to a recipient from year-to-year for philanthropic funding and the NSF. We find that when we order recipients by amount received from the NSF in 2018 there is a Spearman Rank Correlation of 0.95 with the ordering in 2019. This is greater than 98.4% of philanthropic funders who gave to at least 10 science recipients. Furthermore, for the philanthropic recipients, the mean Spearman Rank correlation was only 0.24 between the order of recipients in 2018 and 2019. We believe that the greater variation in amounts from philanthropy potentially arise from major one-time gifts or dedications at an institution which are more likely to change their ordering of top recipients. In contrast, total federal funding is often the aggregate result of grants from numerous PIs such that total funding to institutions remains largely static in time.

IX. Analyzing Traditional Science Funders

To further enrich our analysis, we analyzed a subset of donors that reflect traditional science funders who often solicit applications to fund specific projects. To identify such funders, we used the 15 US-based nonprofits who are members of the Science Philanthropy Alliance and an additional 12 other major funders known to solicit proposals directly from scientists (see Table S1).

A key question is whether these traditional science funders are less locally focused due to their emphasis on identifying worthy scientific recipients. A few examples of the geographic distribution of funding from these traditional funders are shown in Fig. S9. We see that, despite national solicitations, all funders have a significant focus on local communities with 14.5% of grants given locally and 30.1% of funds given locally. These numbers are about half of the locality bias across all funders, but still well above our random baseline of 4.5% of grants and dollars given locally (Fig. S19).

We can further examine a funder's locality bias by comparing patters in random giving networks. In Table S1, we show the actual fraction of funds given locally compared to the mean fraction of funds given locally across 100 randomized sets of recipients. We find that for most of the funders there was never a randomization with greater locality bias than in the data. For a few large foundations however, the bias was not statistically significant, such as for the Templeton Foundation in Pennsylvania and the Charles Koch Foundation in Kansas.

X. Donor Overlap of Recipients and Link Prediction

To consider the frequency of shared donors we focused on grants in the year 2019 and limited to funders who were not donor advised funds, sports conferences, or other similar institutions

(see Sec. V above). We also removed subgrants (grants between two nodes who both received more grant than they gave) or between donor organizations (organizations who both gave more grants than they received), giving us a completely bipartite network with 23,938 donors and 9,120 recipients with 71,973 edges.

We began by determining the Robins-Alexander clustering for bipartite networks to obtain a coefficient of 0.0448, which we can compare to the bipartite network density of 0.00033 (number of edges divided by number of donors times number of recipients).

We next explored the presence of three and four node motifs in this network as explained in the main text. We benchmarked the presence of each motif to the mean of its appearance in 100 randomizations using the bipartite configuration model. We found that while the real network had 1,501,473 four cycles (the last motif on the x-axis in Fig. 4a), for a degree-preserving null model that motif occurred on average only 635,500 times across the randomizations (58% less).

To further verify that the four cycles are not solely due to locality, we also considered a null model that preserves both degree and proximity. We did so by ensuring that links from a funder to a recipient in the same state, remained so after shuffling. Specifically, for each state, we build stublists of donors and recipients based on their in-state giving or receiving and shuffled from those lists. We then shuffled the remaining links where funders and recipients were in different states as well. We found that preserving both proximity and degree led to an average of 786,525 four cycles across 100 randomizations. Notably this is 23% higher than the number of four cycles when proximity was not preserved, yet still 48% lower than the number of four cycles in the real network, demonstrating that while locality plays a role in four cycles, it cannot fully explain the effect.

We next carried out an explicit prediction for grants in 2019 using the funding network from 2018. We began by limiting the grant network of 2018 containing 29,173 donors to 17,154 donors who also gave grants in 2019. Similarly, we restricted to recipients who received from at least 5 foundations and who also appeared in 2019, giving us 3,279 recipients. We then calculated the bipartite Adamic-Adar (AA) Index for each possible edge as in Davis et al.[32] using the grants network from 2018. We converted the raw score of the AA index for each edge to a prediction score using the hyperbolic tangent of the raw score. If the edge actually existed in the grant network from 2019, we labeled the test score as 1 and if it did not we labeled the test score as 0. This then allowed us to calculate the performance metrics for our predictions.

We considered various thresholds for the grants amounts for our predictions, ranging from $1 to $10k, we find that the area under the receiver-operator curve AUROC was quite consistent within this range ranging from 0.87-0.9 depending on the value. While our AUROC score is quite good (demonstrating the strong overlap among funders), we also considered the area under the precision recall curve (AUPRC), which often provides a better measure when there are few positive samples (most possible links in the network do not appear). We find that for a threshold of $10k the AUPRC is 0.19. The baseline of AUPRC is the fraction of positive edges in the network (or more directly, the network density), and for this case the ratio of the AUPRC to network density is 87, meaning that using the AA index there is an 87x improvement in prediction over random prediction of grants. This is comparable to the best observed ratios of AUPRC in other prediction contexts.[33]

XI. Paper Acknowledgements and Philanthropic funding

To further understand philanthropic funding, we also tested if we could identify philanthropic funders in paper acknowledgements. To do so, we collected funder information through dimensions.ai. We identified 42 US funders in dimensions.ai that were categorized as being of type 'Nonprofit.' Notably, this is considerably fewer funders than the thousands we identified as having supported scientific institutions suggesting that most philanthropic funders are never mentioned explicitly in paper acknowledgements. For the 42 funders that did appear, we identified 21,472 papers that acknowledged them. In Fig. S22 we show the topic areas of these papers, finding that most philanthropic support went for papers in Medicine/Health or Biology.

XII. Data Availability

As noted in the main text, there is a need for research and measurement of philanthropic giving to science. Given the increasing role of this source of support, we believe it is crucial for researchers to better quantitative understandings of the non-profit ecosystem. All data and code for replicating our results are available at https://github.com/Barabasi-Lab/mapping-philanthropy/. There, we provide the relevant grants networks, including giver, recipient, amount, and stated purpose. We also include organization metadata about the givers and receivers (nodes) including name, EIN, location (city, state, zipcode), NTEE code, assets, expenses, and revenue. Also included is a list of the public and other universities that we included manually despite them either not having an NTEE code or not having an NTEE code corresponding to our original definition of science. We hope that this directory will serve

to jumpstart research in this area and improve our understanding of philanthropic funding of science.

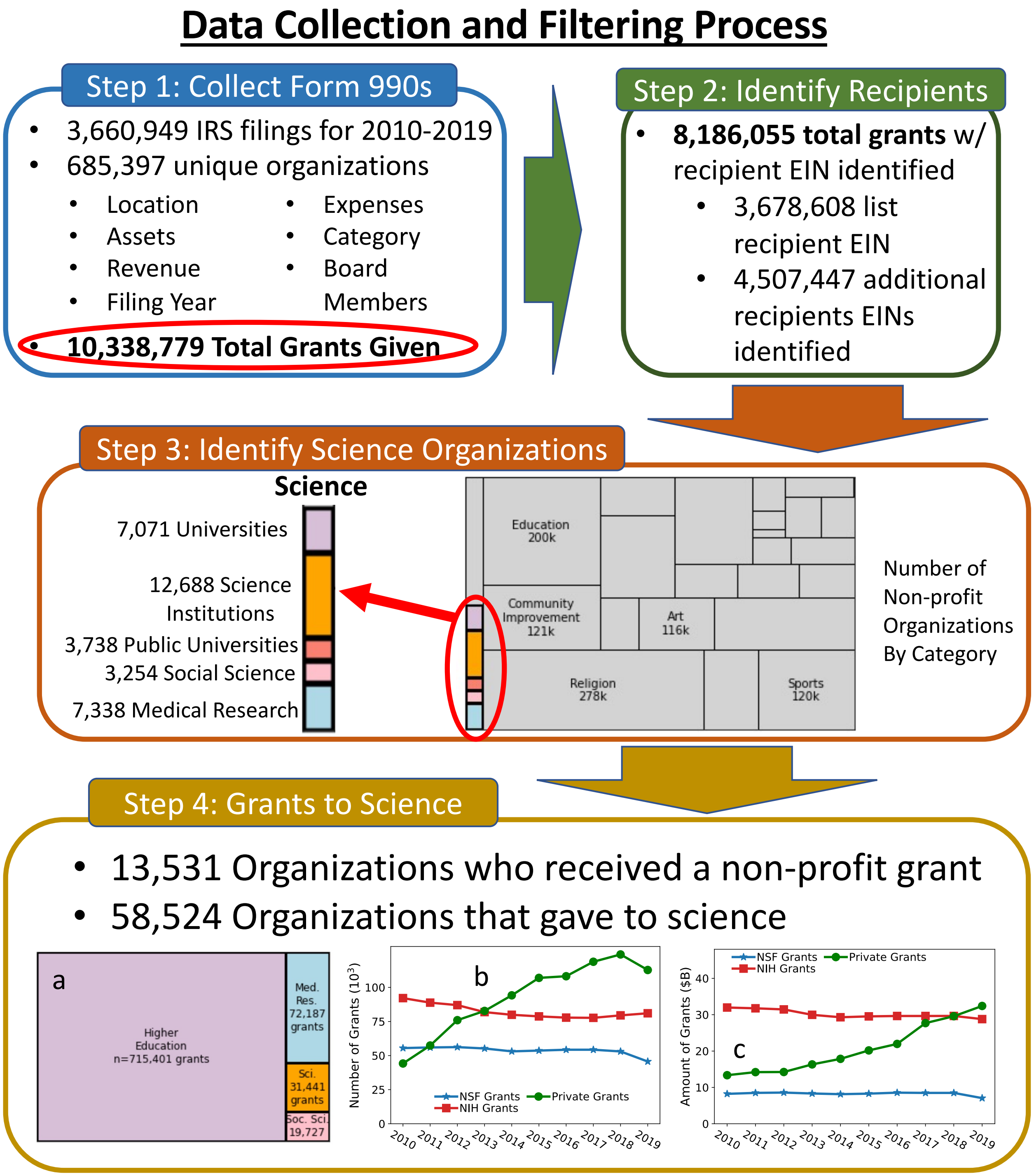

Fig. S1. **Data Collection and Processing**. In Step 1 we collect over 3.6M nonprofit tax filings and extract information on finances, category, location, and grants given by these organizations. Missing Employer ID Numbers (EIN) of grant recipients are identified in Step 2 and then the subset of organizations involved in Science is determined in Step 3. In Step 4, grants to scientific organizations (from Step 3) are selected. (**a**) The majority of scientific grants are to higher education (including public universities). Both the number (**b**) and amount (**c**) of grants from the nonprofit sector surpass giving by the NSF and is commensurate with NIH funding.

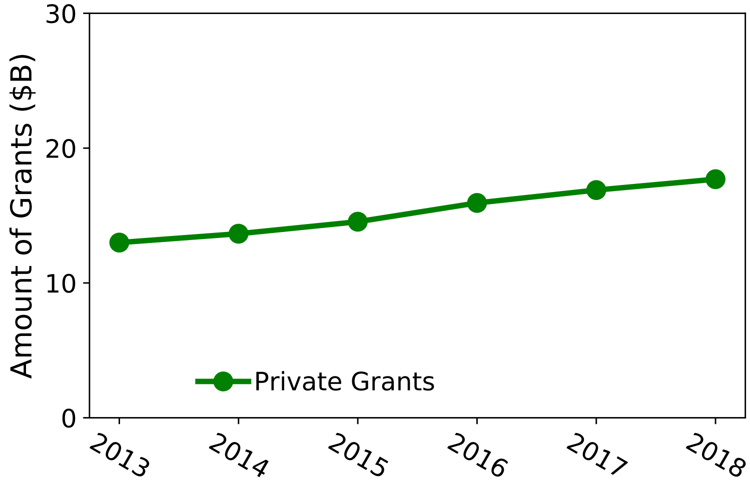

Fig. S2. **Increase in Philanthropic Grant Amounts to Science**. The amount given by the subset of organizations that reported in all years from 2013-2018.

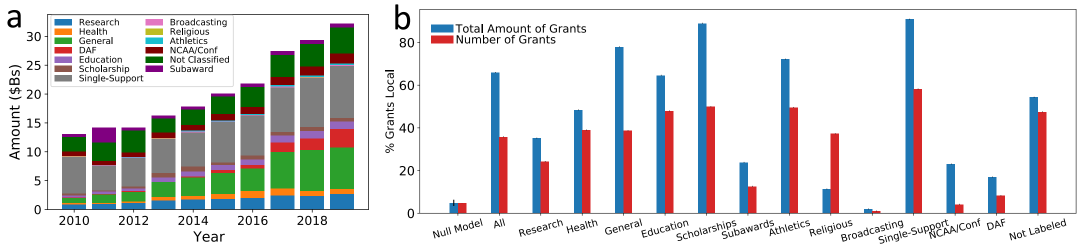

Fig. S3. **Grant purposes**. **(a)** The amount of grant dollars to universities and science institutions that had a stated purpose grouped under specific categories. Funds specifically mentioning research reached $2.7B in 2019 and funds mentioning health reached $1B. Single-support foundations and affiliated hospitals (also included in the 'single-support' category) represented $9.0B in funding for universities and science institutions. **(b)** Across each of our different grant categories, we show the fraction of dollars and grants that went in the same state as the donor. Research funds tend to be somewhat less local than scholarships, general, or education funding, but still far more local than random. Broadcasting grants primarily come from the Corporation for Public Broadcasting which distributes grants throughout the nation leading them to be less local.

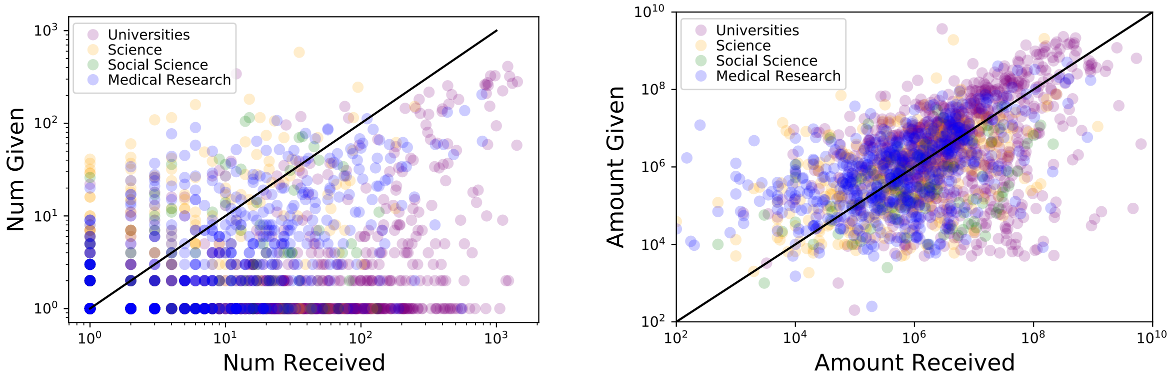

Fig. S4. **Classifying Givers and Receivers**. For the 2,380 organizations that both gave and received grants, we show the number of grants given vs received (left) and the total dollar amount of grants given vs received (right). The x=y line (black) provides a binary classification when necessary.

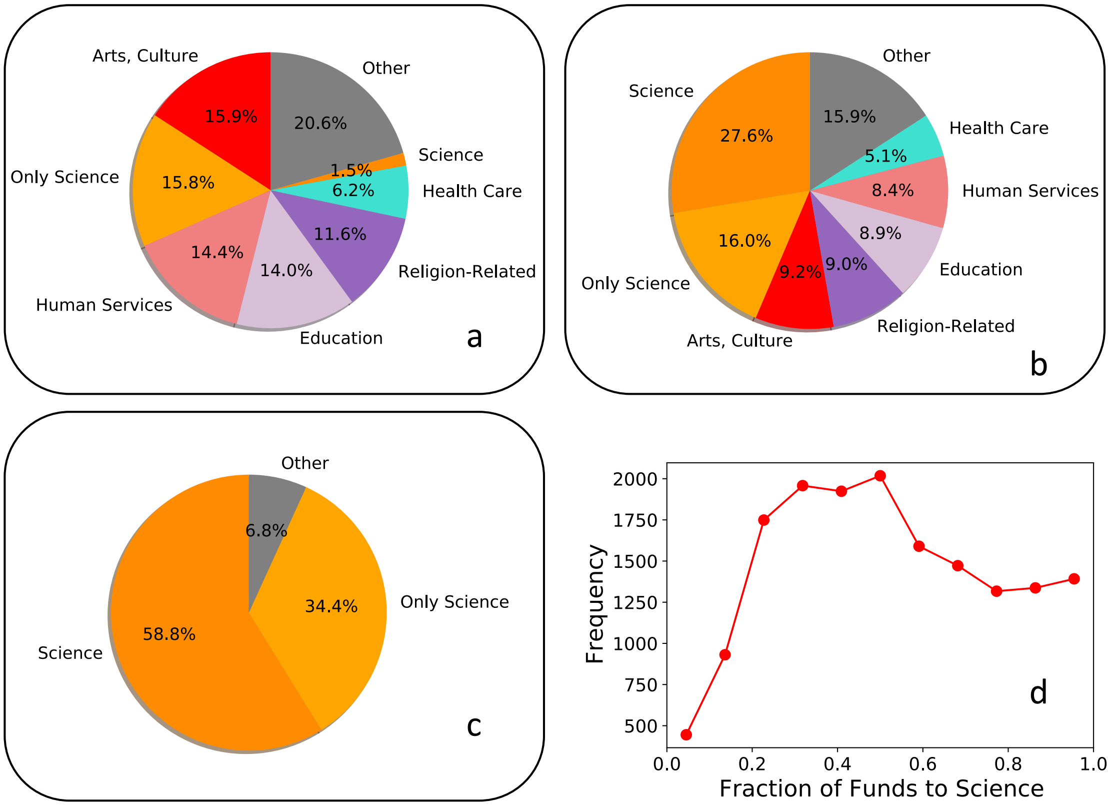

Fig. S5. **Other focuses of philanthropic funders of science**. (a) The distribution of primary granting subject for organizations that gave at least one grant to science reflects the varied interests of non-profit funding organizations. (b) The primary funding subject areas by dollar amount for organizations that gave at least one grant to science. (c) The fraction of total funds to science contributed by givers with a particular focus area. Givers whose main beneficiary was science contributed 59% of the total funds to science while 34% of funds to science came from those who gave exclusively to science. Givers with a main focus outside of science made up only 7% of the total funding to science. (d) For the givers whose primary beneficiary was science, we show what fraction of their funds went to science and what fraction was divided among other areas.

a

Rank	Giver Name	Number of Grants
1	GATES FOUNDATION	6533
2	SHELL OIL COMPANY FOUNDATION	6423
3	ERNST & YOUNG FOUNDATION	3746
4	CORPORATION FOR PUBLIC BROADCASTING	3418
5	THE PRUDENTIAL FOUNDATION	3126
6	JOHN TEMPLETON FOUNDATION	3046
7	UNITED NEGRO COLLEGE FUND INC	2468
8	MOORE FOUNDATION	2448
9	ROBERT WOOD JOHNSON FOUNDATION	2375
10	THE FORD FOUNDATION	2290
12	AMERICAN CHEMICAL SOCIETY	2257
13	CYSTIC FIBROSIS FOUNDATION	2189
14	MACARTHUR FOUNDATION	2189
15	HEWLETT FOUNDATION	1957

b

Rank	Giver Name	Amount Given
1	GATES FOUNDATION	6.5B
2	HEWLETT FOUNDATION	1.2B
3	CORPORATION FOR PUBLIC BROADCASTING	1.1B
4	AMERICAN HEART ASSOCIATION INC	1.1B
5	LILLY ENDOWMENT INC	1.1B
6	MOORE FOUNDATION	942M
7	AMERICAN CANCER SOCIETY INC	882M
8	CYSTIC FIBROSIS FOUNDATION	755M
9	SUSAN THOMPSON BUFFETT FOUNDATION	716M
10	PACKARD FOUNDATION	698M
11	JOHN TEMPLETON FOUNDATION	684M
12	THE PEW CHARITABLE TRUSTS	639M
13	FRED HUTCHINSON CANCER RESEARCH CENTER	505M
14	THE FORD FOUNDATION	497M
15	MACARTHUR FOUNDATION	488M

c

Rank	Recipient Name	Number of Grants
1	UNIVERSITY OF PENNSYLVANIA	7522
2	STANFORD UNIVERSITY	7494
3	UNIVERSITY OF MICHIGAN	6772
4	YALE UNIVERSITY	5799
5	DUKE UNIVERSITY	5582
6	JOHNS HOPKINS UNIVERSITY	5442
7	OHIO STATE UNIVERSITY	5113
8	NORTHWESTERN UNIVERSITY	4686
9	UNIVERSITY OF COLORADO	4599
10	HARVARD UNIVERSITY	4556
11	UNIVERSITY OF CHICAGO	4413
12	CORNELL UNIVERSITY	4405
13	DANA-FARBER INSTITUTE	4372
14	NEW YORK UNIVERSITY	4352
15	MEMORIAL SLOAN KETTERING	4285

d

Rank	Recipient Name	Amount Received
1	UNIVERSITY OF FLORIDA	7.5B
2	DUKE UNIVERSITY	6.4B
3	HARVARD COLLEGE	5.4B
4	GEORGIA INSTITUTE OF TECHNOLOGY	4.8B
5	NEW YORK UNIVERSITY	4.7B
6	UNIVERSITY OF WISCONSIN	3.9B
7	INDIANA UNIVERSITY	3.3B
8	STANFORD UNIVERSITY	3.1B
9	UNIVERSITY OF NEBRASKA	2.9B
10	UNIVERSITY OF ILLINOIS	2.6B

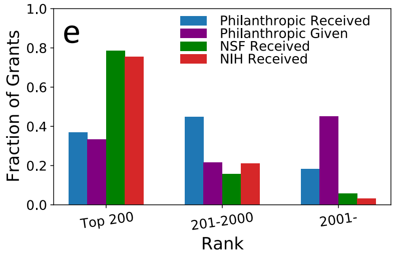

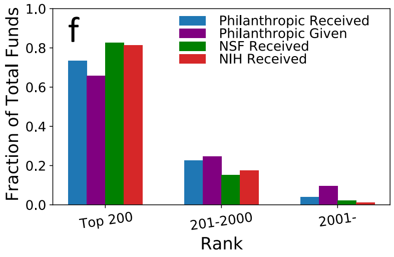

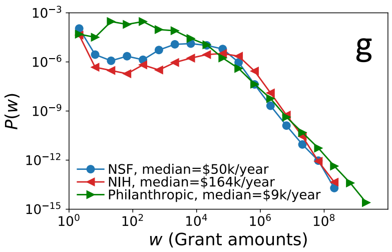

Fig. S6. **Number and amount of grants**. The top givers by (a) the number of grants, and (b) total amount given over 10 years. Donor advised funds, community foundations, single-support foundations, and universities acting as redistributors were excluded from these lists. The top recipients by (c) number of grants and (d) total amount received over 10 years. The ranking by amounts is biased by single-support foundations. (e) The fraction of grants given or received by top givers/receivers compared to lower ranked givers and receivers. We see that NSF and NIH giving is more concentrated among a select group of fewer than 200 institutions, with philanthropic funding sources and recipients being more broadly distributed. Especially philanthropic funders are seen to be quite abundant. (f) The distribution of total funds given by top givers/recipients compared to smaller non-profits. (g) The distribution of NSF, NIH, and Philanthropic grant amounts.

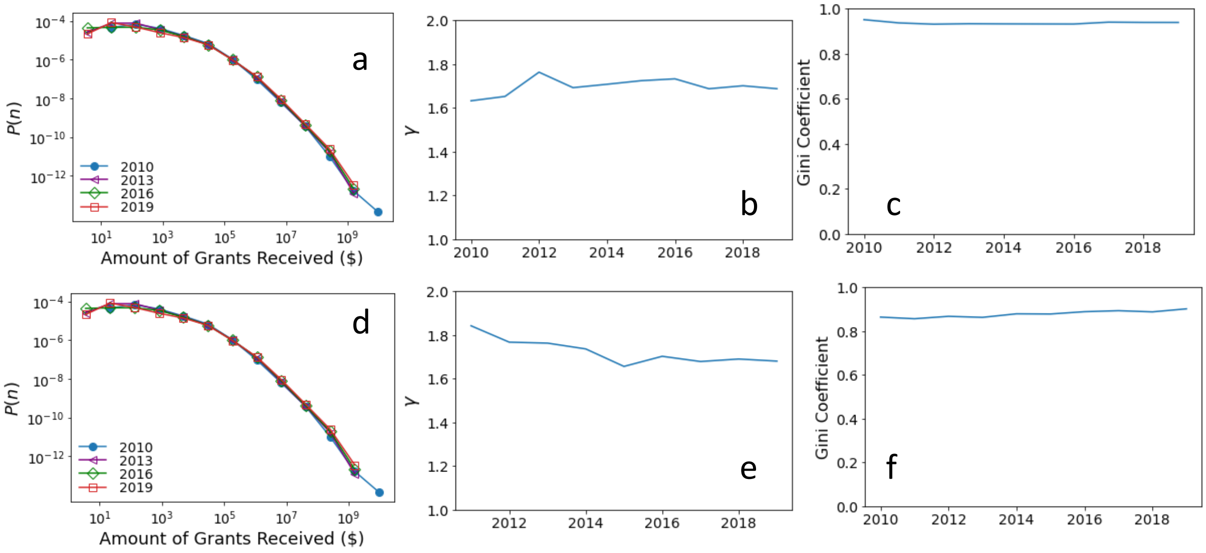

Fig. S7. **Distributions of grants and inequality for philanthropic funders**. (a) the distribution of amount of philanthropic funding received for different years. (b) The power-law exponent of the distribution of philanthropic funds for over time each year. (c) The Gini Coefficient across total amount of funds received by different institutions over time. (d)-(f) Same as (a)-(c) but limited to only include research specific funding (funding that either mentions 'research' in the purpose or comes from a known research funder).

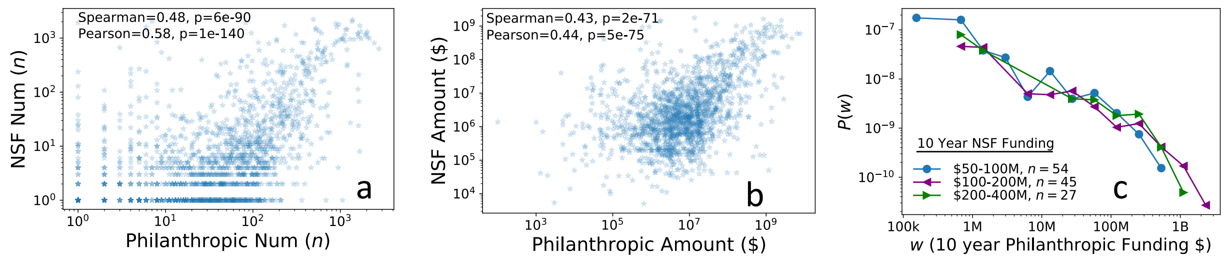

Fig. S8. **NSF and philanthropic grants to individual institutions**. The correlation between philanthropic grants and NSF grants at the level of individual institutions based on (a) number of grants and (b) total dollar amount of grants, from 2010-2019. (c) The distribution of philanthropic funding for a given level of NSF funding.

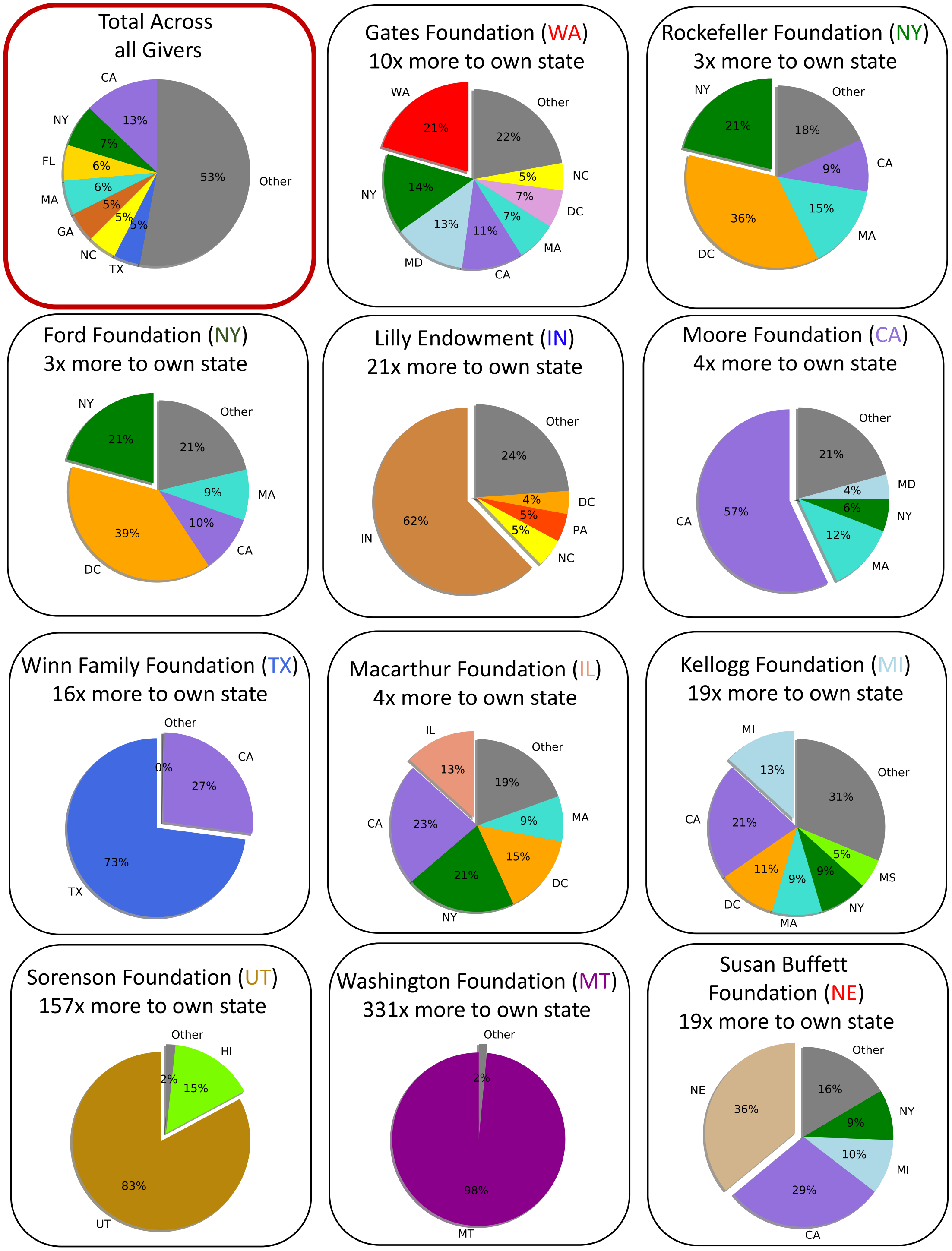

Fig. S9. **Examples of local grant giving**. The spatial distribution of funding across all givers (top left) and the distribution for several example foundations. The first 8 foundations are traditional funders of research (SI, Sec IX).

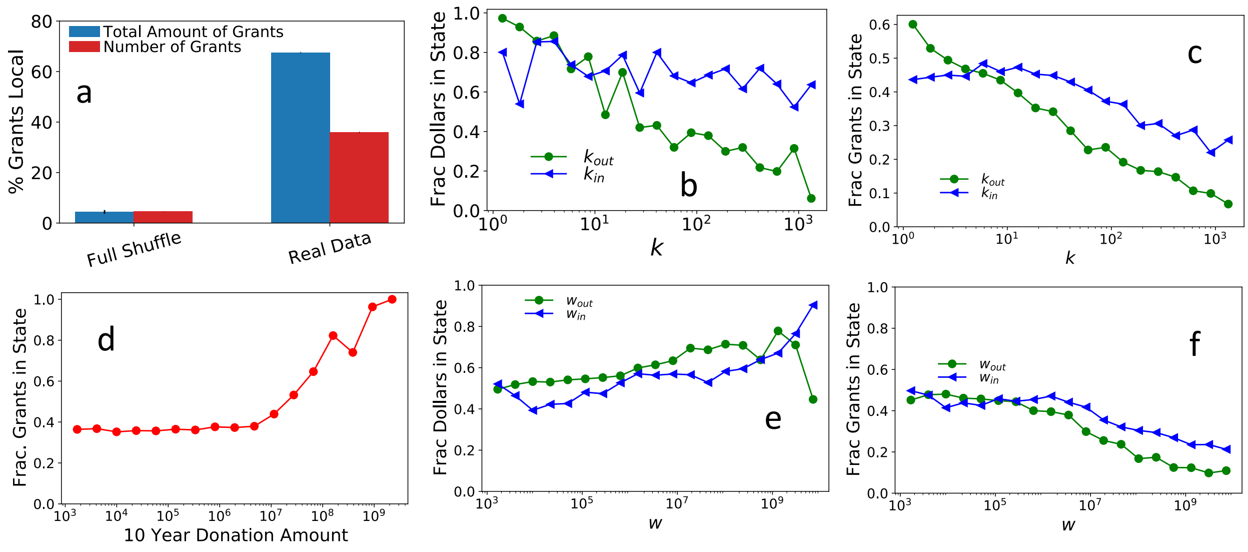

Fig. S10. **Locality of grant giving**. (a) The fraction of grants and dollar amounts given within the same state compared to a randomly shuffled null model. (b)-(c) The fraction of dollars (b) and grants (c) that remained within the state. Only those organizations with at least 10 grants are included. (d) The fraction of grants that are local vs the total 10-year donation amount between the grantor and receiver. Donations above ~$1M/year have an increasing likelihood to be to a local recipient. (e)-(f) The fraction of (e) dollars and (f) number of grants given within the same state for givers (green) and recipients (blue) by dollar amount, *w.*

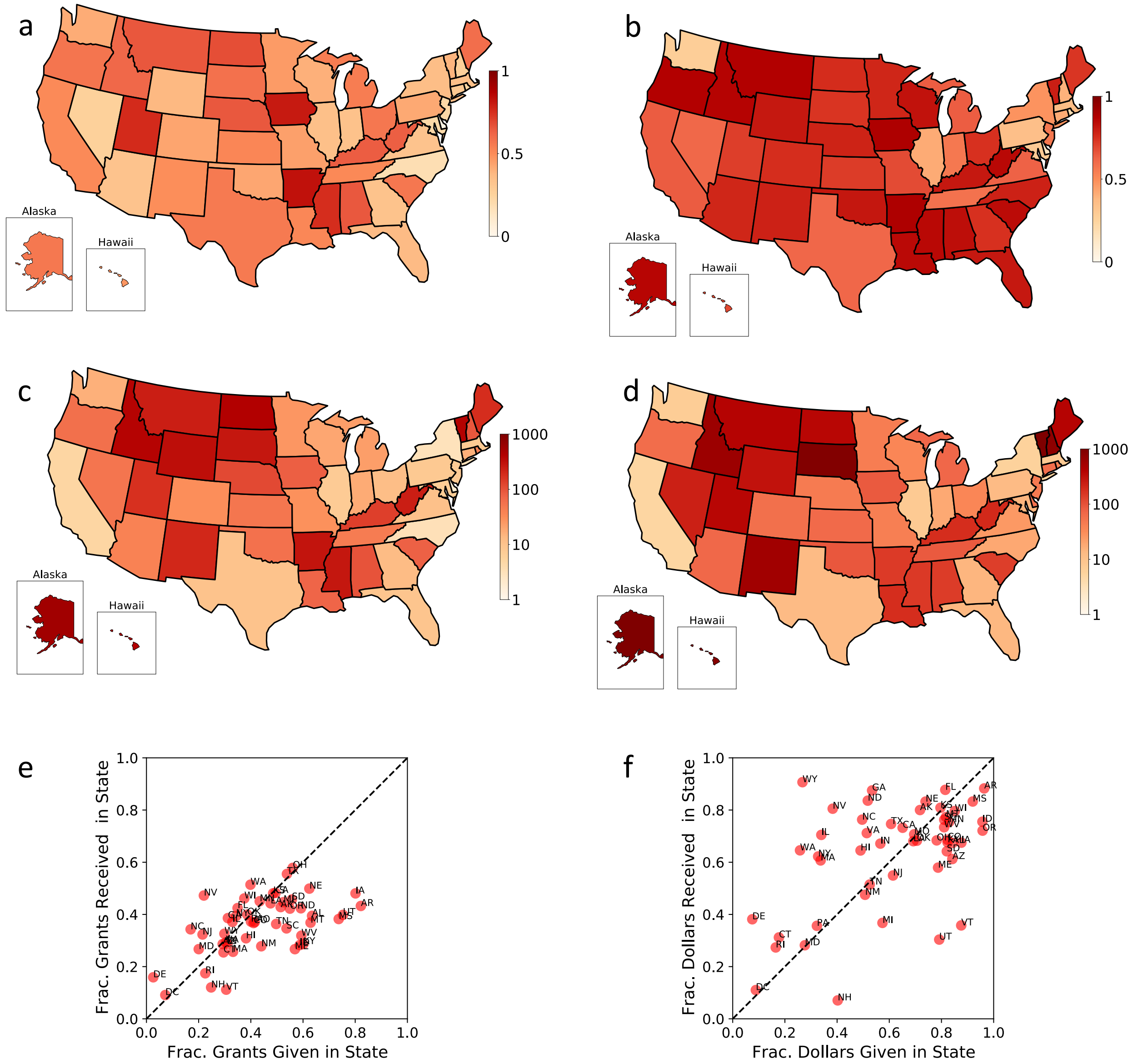

Fig. S11. **Local Giving and Receiving by State**. Choropleths of (a) the fraction of grants and (b) the fraction of dollars given in state by donors located in the same states. In (c) and (d) we normalize these amounts by the fraction of total grants or dollars given to the state such that 1 reflects donors in a state giving the same proportion as donors overall. (e) The fraction of grants given by funders in their home state versus the fraction of grants received by recipients within the state from the state. States further to the right have local funders who support them, whereas those further to the left have local funders giving elsewhere. Below the line are funders who despite having strong local giving, still receive more funding from outside the state meaning the local funding is proportionately insufficient. (f) Same as (e) but based on the total dollars rather than number of grants.

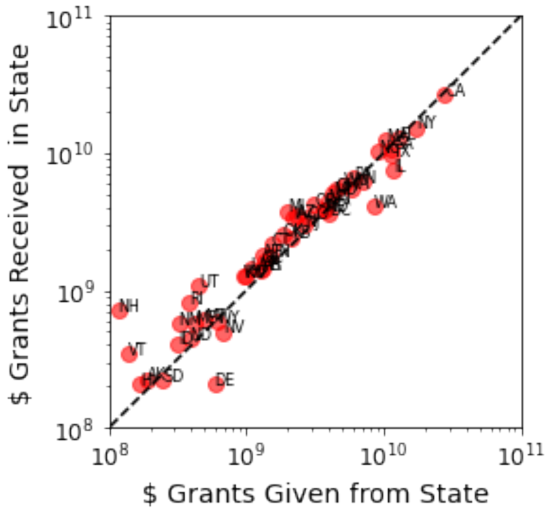

Fig. S12. **Local Giving and Receiving by State**. The Amount of grants originating from each state (given by a foundation registered in that state), versus the amount of grants received in a state. We find that the two are nearly perfectly correlated, with most deviations occurring for states with low levels of grants given and received.

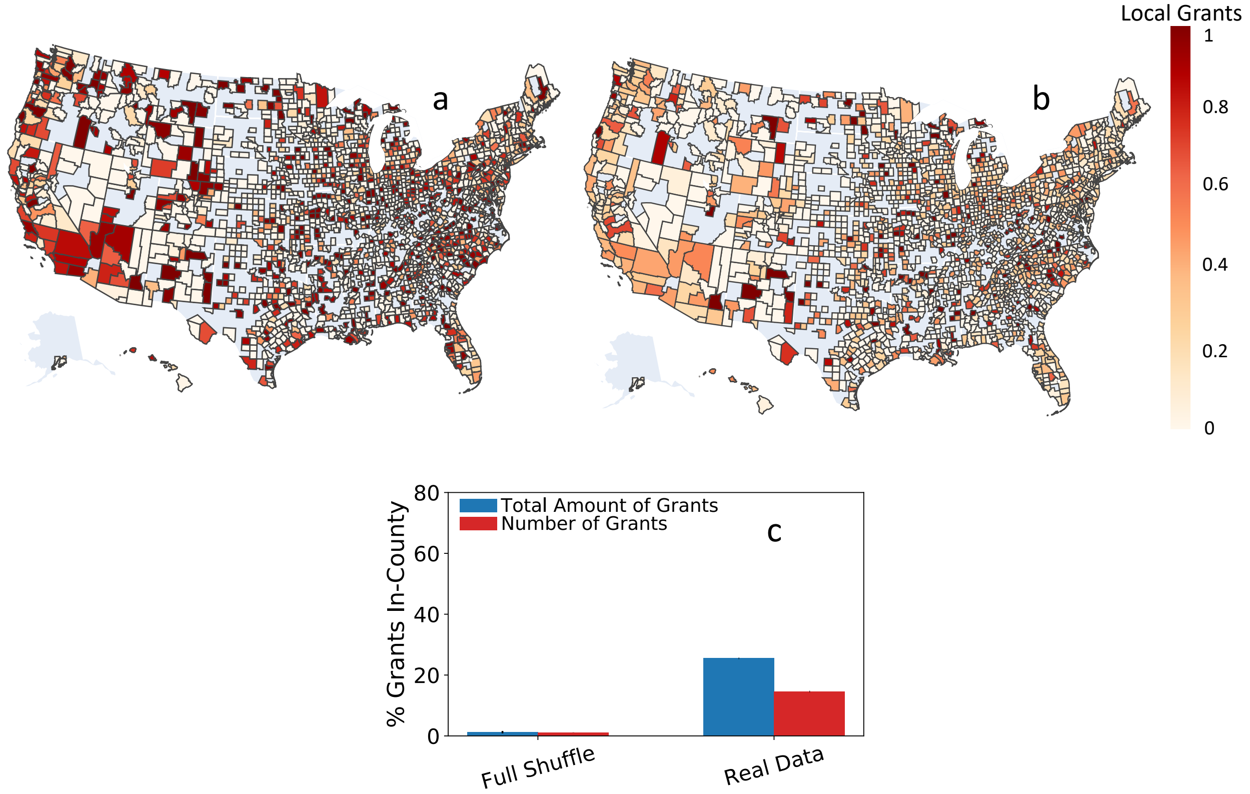

Fig. S13. **Locality by county.** (a) The fraction of dollars given within the county for funders in particular counties. (b) The fraction of grants given locality by county. (c) Comparing in-county giving for both the number and amount of grants to the null model where the number of grants given and received is preserved but the sources are shuffled.

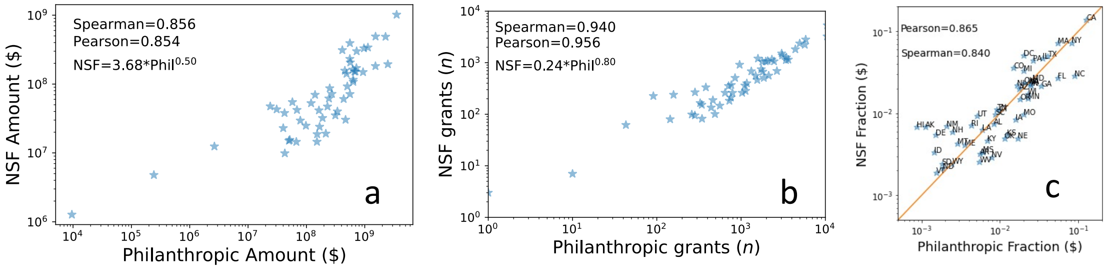

Fig. S14. **Correlations of NSF and Philanthropic funding across states**. Panel (a) shows the correlation between the total amount from the NSF versus philanthropic sources to particular states and territories, while (b) shows the correlation for the total number of grants. In both cases we observe very high correlations, suggesting that the same states tend to receive greater funding from both the NSF and philanthropy. (c) Same as (a) but only including continental US states and normalized by the total NSF and philanthropic funding.

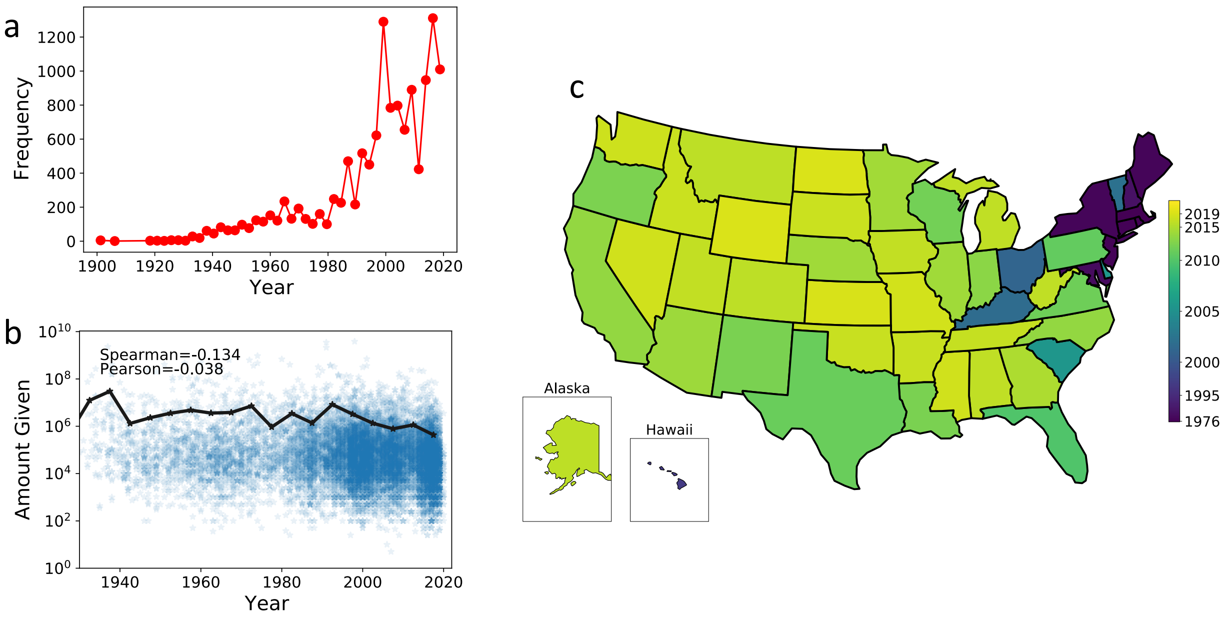

Fig. S15. **Foundation Age**. (a) For the 12,984 donors who provided a ruling year (year when the IRS established the donor's exempt status) we show the frequency that donors had a particular ruling year. We see that most of the foundations in the sample are fairly recently formed (less than 20 years). (b) We plot the amount given by the foundation versus the ruling year. We see that older foundations are somewhat more likely to be giving larger amounts, the effect sizes are fairly moderate. (c) We show for different states the median ruling year of donor organizations weighted by their total amount given. We see that in many of the Northeastern states there exist more older nonprofit donors that bring the median ruling year down to the late 1990s or early 2000s in these states.

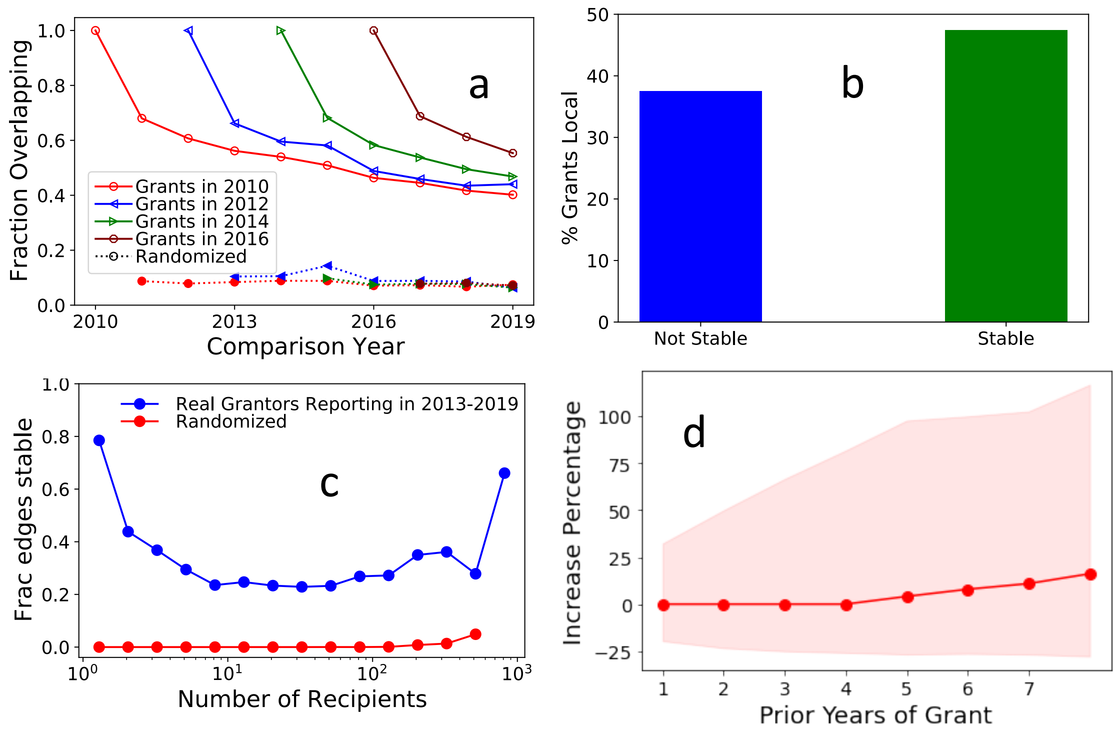

Fig. S16. **Continuing grants in time**. (a) For grants beginning in a particular year (2010, 2012, 2014, 2016), we show the fraction of grants that repeat in future years. This is compared to a randomized degree-preserving null model. (b) The fraction of stable edges based on the number of grants given by the grantmaker. Grantmakers who give fewer grants are more likely to give in a stable manner rather than grantmakers who give more grants. The later increasing for donors giving to over 100 recipients, likely reflects the limited number of major science recipients and also arises in the randomized versions of the granting network. (c) Stable grants are slightly more likely to be local, but that the difference is fairly moderate in terms of effect size. (d) Comparison of the percentage increase from year-to-year in philanthropic funding for grants that repeat. Compared to Fig. 4f in the main text, here we show the relative, rather than absolute increase. We see again that while the median increase is still zero, at the third quartile the increase can approach nearly double the previous year. This will still be of roughly the same order of magnitude as the previous donation, which is worth noting given the scale-free distribution in donation amounts.

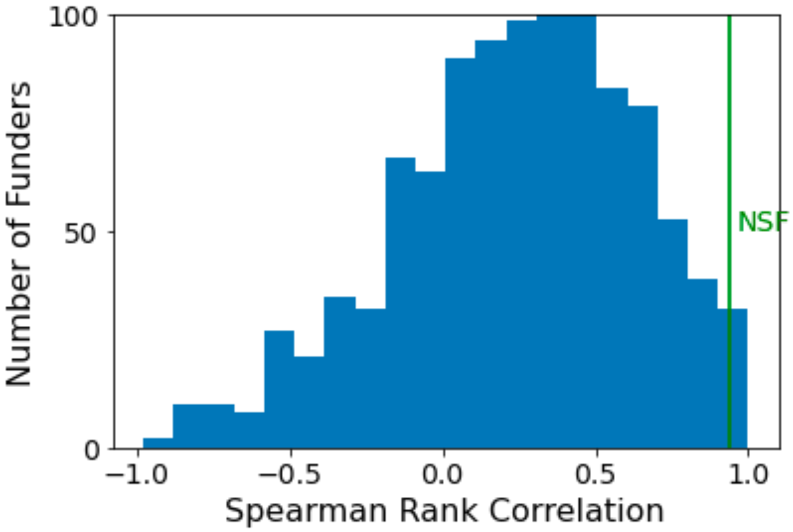

Fig. S17. **Philanthropic grants and NSF grants amount rank stability**. Distribution of the Spearman Rank correlation between the amounts given to recipients in 2018 by philanthropic funders and the amounts given to recipients in 2019 restricted only to funders with at least 10 distinct recipients in both 2018 and 2019.

a

Rank	Recipient Name	Amount Received
1	STANFORD UNIVERSITY	2.3B
2	JOHN HOPKINS UNIVERSITY	1.6B
3	HARVARD UNIVERSITY	1.3B
4	MIT	1.2B
5	CLIMATEWORKS	1.2B
6	DUKE	1.0B
7	PENN	953M
8	UNIVERISTY OF CALIFORNIA SYSTEM	946M
9	UNIVERSITY OF MICHIGAN	940M
10	BROAD INSTITUTE	869M

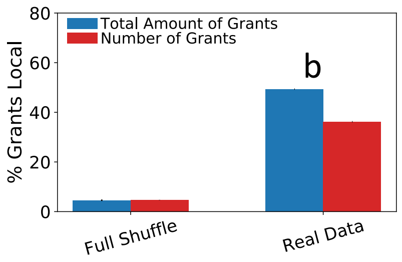

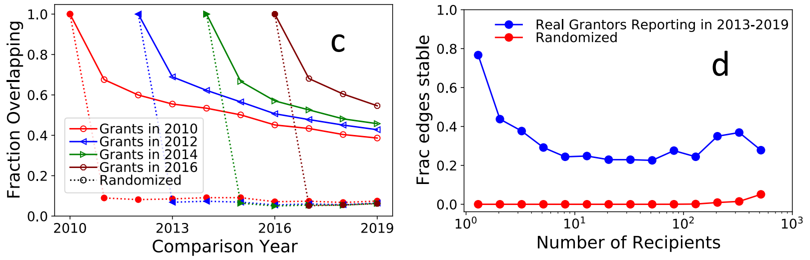

Fig. S18. **Results without special cases (single-support foundations, DAFs, and Sports Conferences)**. Our results are qualitatively similar even after removing single-support foundations and other university affiliates (such as hospitals). (a) The composition of the list of top recipients by amount is considerably changed due to the removal of single-support foundations and affiliated hospitals, though many of the same institutions remain on the list. In (b) we show that there continues to remain a strong local effect. The fraction of dollars given locally is reduced (from 67% to 49%), but still remains far above the random baseline. The fraction of grants given locally remains essentially unchanged. (c) The level of stability in grant-giving remains similar to before and (d) the effect of the number of recipients on stability also continues to be observed.

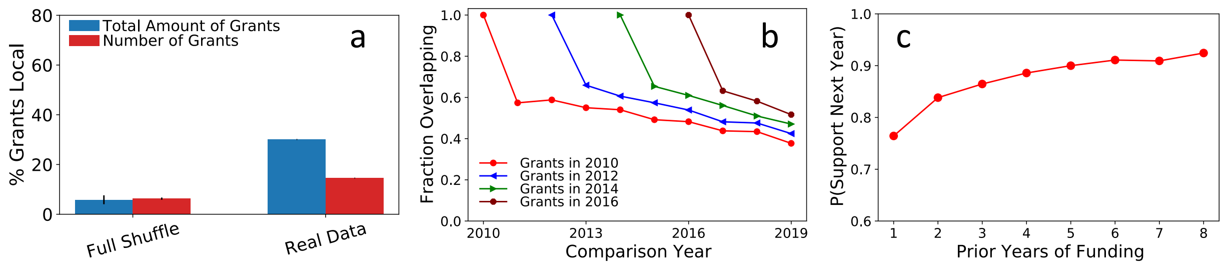

Fig. S19. **Traditional science funders**. Our results are similar for a set of traditional science funders. (a) The locality bias is reduced for these funders, but still noticeably present. Traditional funders gave 30% of their funds and 15% of their grants in-state, less than other philanthropic donors, but still far above the random baselines of 5% of funds and dollars. (b) The stability of funding is generally similar for traditional science funders. (c) Persistence is present for traditional funders as well.

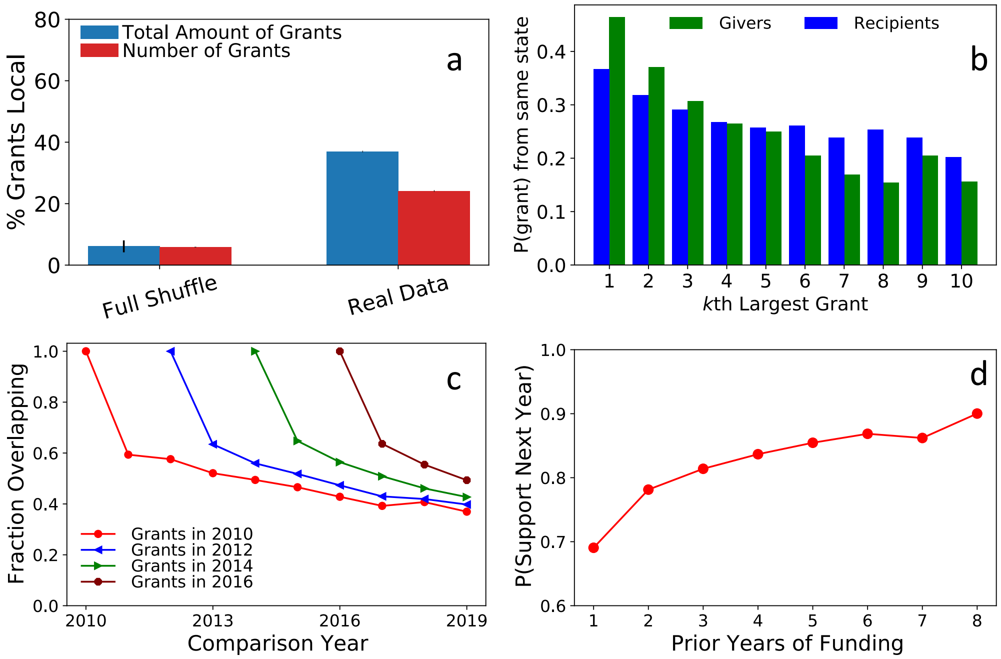

Fig. S20. **Funds categorized as research or health, or that came from a traditional science funder**. Our results remain similar when we combine funds with a purpose that mentions 'research' together with grants by traditional science funders. (a) The locality bias for this set of grants and funders remains present. (b) For this set of funders and grants, a funder's largest grant is still local considerably more often than their comparatively smaller grants. (c) Grants continue to have high rates of stability and repetition from one year to the next. (d) The grants for research continue to have increasing persistence as more years of prior funding still lead to a greater likelihood to continue the grant.

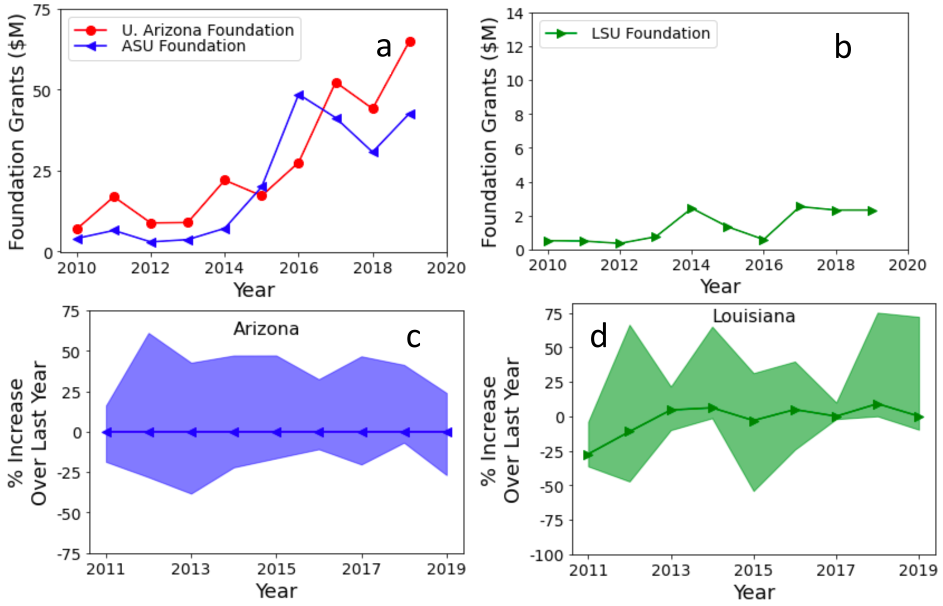

Fig. S21. Philanthropy and cuts to higher education. Amount of foundation grants to public universities in Arizona and Louisiana, states with major cuts to higher education. (a) Foundation grants to the University of Arizona and Arizona State University affiliates increased over the years (though it must be recalled that the increase is exaggerated due to more filers starting to file online), while (b) grants to the LSU Foundation remained considerably lower and constant over the years. Furthermore, for each supporter of a public university affiliate in Arizona (c) or Louisiana (d), we examined how their support in one year compared to their support the previous year. Across all donors who supported the university in the previous year, we plot the median percent increase in their support, and shade the interquartile range. We find that for the Arizona public universities, the median increase in support is always 0%, meaning the amounts remained the same, and that for Louisiana there is greater variation though we still that funders did not tend to increase their support in a consistent manner from year to year.

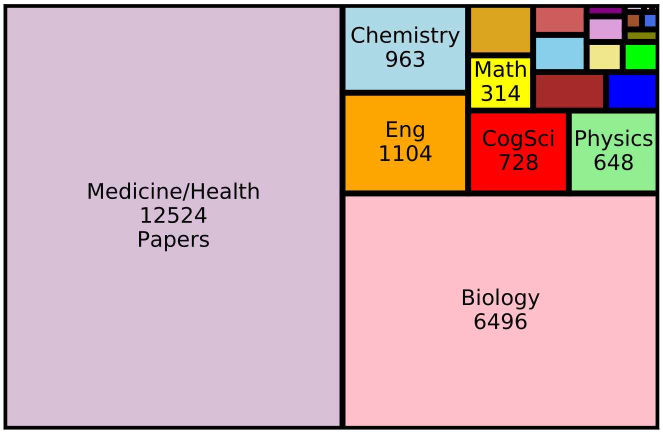

Fig. S22. **Philanthropic Support by Field**. For grantors listed as non-profits in the Dimensions.ai database, we examine the set of 21,472 papers associated with their funding. We find that the majority of these papers are in the categories of Medicine/Health and Biology, with fewer numbers in Engineering (Eng), Chemistry, Cognitive Science (CogSci), Physics, and Math.

Table S1. List of Traditional Science Funders

Foundation	State	Fraction of Dollars Local in data	Mean Fraction of Local Amount in Randomizations	Fraction of Randomizations with more local dollars than data
ALBERT & MARY LASKER FOUNDATION	NY	0.002	0.059	0.5
ALFRED P SLOAN FOUNDATION	NY	0.121	0.08	0
HEISING-SIMONS FOUNDATION	CA	0.438	0.078	0
MOORE FOUNDATION	CA	0.57	0.075	0
THE KAVLI FOUNDATION	CA	0.288	0.071	0
PACKARD FOUNDATION	CA	0.748	0.074	0
HOWARD HUGHES	MD	0.042	0.017	0.02
JOHN TEMPLETON FOUNDATION	PA	0.049	0.043	0.19
LEON LEVY FOUNDATION	NY	0.754	0.08	0
THE LYDA HILL FOUNDATION	TX	0.002	0.039	0.06
THE ROCKEFELLER FOUNDATION	NY	0.212	0.079	0
ROSS M BROWN FOUNDATION	CA	0.514	0.066	0
SERGEY BRIN FOUNDATION	CA	0.789	0.094	0
SHANAHAN FAMILY FOUNDATION	CA	0.764	0.084	0
WINN FAMILY FOUNDATION	TX	0.729	0.044	0
GATES FOUNDATION	WA	0.205	0.015	0
WKKELLOGG FOUNDATION	MI	0.131	0.023	0
THE FORD FOUNDATION	NY	0.207	0.08	0
MACARTHUR FOUNDATION	IL	0.134	0.03	0
HEWLETT FOUNDATION	CA	0.746	0.078	0
CHARLES G KOCH FOUNDATION	KS	0.003	0.006	0.54

ROBERT WOOD JOHNSON FOUNDATION	NJ	0.021	0.015	0.22
LILLY ENDOWMENT	IN	0.622	0.02	0
THE PEW CHARITABLE TRUSTS	PA	0.229	0.036	0
SUSAN KOMEN BREAST CANCER	TX	0.159	0.034	0
MELLON FOUNDATION	NY	0.169	0.077	0
KNIGHT FOUNDATION	FL	0.133	0.02	0

Table 1. **Locality of large foundations**. The fraction of dollars given local in the data, the mean fraction of local dollars over 100 randomized networks and the fraction of realizations that had greater locality than the data (p-value).
